# The Burden Cancer-Related Deaths Attributable to High Body Mass Index in a Gulf Cooperation Council: Results from the Global Burden of Disease Study 2019

**DOI:** 10.1007/s44197-024-00241-5

**Published:** 2024-05-13

**Authors:** Majed Ramadan, Rbab M. Bajunaid, Sereen Kazim, Noara Alhusseini, Ali Al-Shareef, Nourah Mohammed ALSaleh

**Affiliations:** 1 Population Health Research Section King Abdullah International Medical Research Center (KAIMRC), King Saud Bin Abdulaziz University for Health Sciences, Ministry of National Guard - Health Affairs, P.O.BOX 9515, Jeddah, 21423 Kingdom of Saudi Arabia; 2 King Abdullah International Medical Research Center (KAIMRC), P.O.BOX 9515, Jeddah, 21423 Saudi Arabia; 3 College of Medicine, Jeddah University, Jeddah, 23218 Saudi Arabia; 4 College of Medicine, Alfaisal University, Riyadh, Saudi Arabia; 5 King Abdulaziz Medical City, Jeddah, Saudi Arabia; 6 King Abdullah International Medical Research Center, College of Medicine, King Saud Bin Abdulaziz University for Health Sciences, 21423 Jeddah, P.O.BOX 9515, Saudi Arabia; 7 Department of surgical oncology, Ministry of National Guard Health Affairs, King Abdulaziz Medical City, 21955 Jeddah, Makkah, P. O. Box: 7633, Saudi Arabia

**Keywords:** Gulf cooperation council (GCC), Body mass index (BMI), Cancer, Disability-adjusted life-years (DALYs)

## Abstract

**Background:**

BMI has been reported to be a major risk factor for the increased burden of several diseases. This study explores the burden of cancer linked to high body mass index (BMI) in Gulf Cooperation Council (GCC) countries and assesses the correlation with Socio-demographic Index (SDI).

**Method:**

Using Global burden of disease (GBD) 2019 data, the authors quantified cancer burden through mortality, DALYs, age standardized mortality rate (ASMR), and age standardized DALYs rate (ASDR) across sexes, countries, cancer types, and years. Spearman’s correlation tested ASMR against SDI. The authors estimated 95% uncertainty limits (UIs) for population attribution fraction (PAFs).

**Results:**

Between 1990 and 2019, all six GCC countries showed increased number of the overall cancer-related deaths (398.73% in Bahrain to 1404.25% in United Arab Emirates), and DALYs (347.38% in Kuwait, to 1479.35% in United Arab Emirates) reflecting significant increasing in deaths, and burden cancer attributed to high BMI. In 2019, across GCC countries, pancreatic, uterine, and kidney cancer accounted for 87.91% of the total attributable deaths associated with high BMI in females, whereas in male, colon and rectum cancer alone accounted for 26% of all attributable deaths associated with high BMI.

**Conclusion:**

The study highlights the significant impact of high BMI on cancer burden in GCC countries. Moreover, the study identifies specific cancers, such as pancreatic, uterine, and kidney cancer in females, and colon and rectum cancer in males, as major contributors to attributable deaths, urging targeted prevention strategies at reducing weight and encouraging physical activity could greatly lessen the impact of diseases in the GCC countries.

**Supplementary Information:**

The online version contains supplementary material available at 10.1007/s44197-024-00241-5.

## Introduction

High body mass index (BMI), defined as 25 kg/m² or greater, is a complex risk factor with significant implications for public health [1]. It substantially elevates the risk of various health complications, making it a focal point in public health discussions. Obesity, a consequence of high BMI, is firmly established as a risk factor for numerous chronic diseases, including type 2 diabetes, cardiovascular diseases, hypertension, and specific types of cancer [2–3]. The global surge in overweight and obesity rates can be attributed mainly to lifestyle shifts stemming from societal and demographic transitions that unfolded over the past few decades. The Gulf Cooperation Council (GCC) countries, oil-producing nations, have experienced profound lifestyle changes due to rapid economic and demographic transformations. Furthermore, the influence of a Westernized lifestyle has permeated these countries, leading to shifts in dietary habits characterized by increased consumption of processed and high-fat foods [4]. These societal shifts have contributed significantly to the rising prevalence of obesity in the GCC countries, highlighting the urgency of addressing this public health challenge.

BMI has been reported to be a major risk factor for the increased burden of disease in 2013 in Bahrain, Kuwait, Oman, and Saudi Arabia [5]. Consistently, High BMI ranked first risk factor associated with many diseases over the period 1990–2017 in Saudi Arabia [6]. Furthermore, Saudi Arabia, Kuwait and Qatar were among the top ten countries with the highest prevalence of obesity worldwide in 2013 and 2019 [7–8]. On the other hand, cancer ranked as the third leading cause of death and premature disability attributable to high BMI worldwide; it is challenging healthcare systems and communities globally [1]. The GCC countries are no exception to this global trend, as they face a rising incidence of cancer and its far-reaching consequences [9–10]. High BMI is not a benign condition; in recent years, research has underscored its role in cancer development and progression. The relationship between high BMI and cancer has been well-documented in the literature [11]. Excess body weight has been implicated in the development of several types of cancer, including breast, colorectal, and endometrial cancer [12–14] The mechanisms of this relationship are multifaceted, involving metabolic, hormonal, and inflammatory pathways. In essence, high BMI creates an environment within the body that fosters the initiation and progression of cancer cells [11, 15, 16].

According to recent studies [11]; 4 to 8% o cancer cases are attributable to excess body weight. Other studies indicate that each 5 kg/m2 increase in BMI was positively associated with cancers of the uterus, gallbladder, kidney, cervix, thyroid, and leukemia [17]. Despite the escalating rates of obesity and its well-established association with various cancers, there is a scarcity of region-specific data exploring cancer attributable to high BMI in the GCC countries [18–20]. Nonetheless, prevalence and impact vary across countries and populations, including gender-based variations. Cancer cases increased by 136% from 1999 to 2015 and are projected to rise by 63% in 2030 in Saudi Arabia [21]. As obesity and cancer rates continue to rise in the GCC countries, research in this domain becomes a basis for identification and mitigation of preventable risk factors for these carcinomas becomes paramount, mitigating the burden of obesity-related cancers, and promoting overall well-being in the region. Therefore, this study aims to utilize data obtained from GBD, to estimate the burden of cancer related death and disability-adjusted life-years (DALYs) attributable to high BMI for GCC countries between 1990 and 2019 using the latest GBD 2019 dataset. Additionally, the authors assessed the correlation between cancer burden and Socio-demographic Index (SDI). The long-term trends of the age-standardized mortality rate (ASMR) were also analyzed. The main objective of this study was to utilize the data and their fluctuations to guide potential strategies in primary prevention, screening, early detection, and treatment of cancer linked to high BMI.

## Method

### Data Source & Search Parameter

In this population-based epidemiological study, the authors used GBD’s 2019. The GBDs consisted of multiple measures of health loss for 282 causes of death, 359 diseases, and 84 risk factors for each of 204 countries and territories, 23 age groups, and both sexes from 1990 to 2019 [22]. The GBD 2019 study obtained data from several data sources, including national vital statistics, cancer registries, verbal autopsy reports, national health surveys, censuses, and published studies. The authors quantified the burden of cancer attributable to high BMI from 1990 to 2019. The interested data were collected from the Global Health Data Exchange (GHDx) tool (https://ghdx.healthdata.org/gbd-2019) and selected “neoplasm and specific cancer types” as the cause, and “high BMI” for risk; “deaths, DALYs” for measurements “1990–2019” for years; and “number and rate” for metrics. Data were downloaded at both sex, SDI, and GCC country levels. We obtained data at all ages and age-adjusted categories.

### Definitions

This study focused on high-risk populations with a BMI of 25 kg/m^2^ and higher aged 20 years or older in high-income GCC countries in the Middle East. The GCC comprises of six countries; Bahrain, Kuwait, Oman, Qatar, Saudi Arabia, and the United Arab of Emirates located in southwest Asia. In 2012 the GCC had a more than 57 million population. They share similar political, religious, cultural, economic, and social backgrounds.

In this study the authors included cancers that have significant evidence associated with high BMI reported by the World Cancer Research Fund (WCRF) [23]. Cancer presented in this study includes malignant neoplasms defined by the International Statistical Classification of Diseases (ICD) by the Tenth Revision (ICD-10) as codes esophageal (C15-C15.9, D00.1, D13.0) colon and rectal(C18-C21.9, D01.0-D01.3, D12-D12.9, D37.3-D37.5), kidney (C64-C65.9, D30.0-D30.1, D41.0-D41.1), pancreatic (C25-C25.9, D13.6-D13.7), gallbladder (C23-C24.9, D13.5), breast (C50-C50.9, D05-D05.9, D24-D24.9, D48.6, D49.3), uterine (C54-C54.9, D07.0-D07.2, D26.1-D26.9), ovarian cancers (C56-C56.9, D27-D27.9, D39.1) and liver cancer (C22-C22.8, D13.4). Also, the authors included additional cancer sites that have recently been suggested to be associated with high BMI but were not listed by WCRF as sufficient [24–26]. This includes thyroid cancer (C73-C73.9, D09.3, D09.8, D34-D34.9, D44.0), Non-Hodgkin Lymphoma (C82-C86.6, C96-C96.9), Multiple myeloma (C88-C90.9), and Leukemia (C91-C91.0, C91.2-C91.3, C91.6, C92-C92.6, C93-C93.1, C93.3, C93.8, C94-C95.9). The authors calculated the incidences of these cancer subtypes utilizing proportions by sex and country level. The exact time interval between the onset and duration of elevated BMI and the manifestation of cancer remains uncertain.

The DALYs is a summary measure that quantifies the overall burden of disease, which represents the sum of years of life lost due to premature death (YLL) and years lived with disability (YLD). One DALY can be regarded as the loss of 1 year in total health [22]. The Socio-demographic Index (SDI) is calculated by GBD study 2019 as a measurement of overall development covering educational level, distributed income, and total fertility rate [27]. The SDI ranges from 0 to 1, with a higher value implying a higher level of socioeconomic development. high SDI (> 0.81), high-middle SDI (0.70 – 0.81), middle SDI (0.61–0.69), low-middle SDI (0.46–0.60), and low SDI (< 0.46).

### Statistical Analysis

The burden estimates are presented regarding the numbers of DALYs, and deaths. ASMR and age standardized DALYs rate (ASDR) are expressed as the rate per 100,000 people. The ASMR and the ASDR were used to quantify the burden of cancer associated with BMI. The attributable fraction of cancer specific incidence, deaths and DALYs related to high BMI was measured using the Population Attributable Fraction (PAF). This is defined as the proportion of all cases of a specific disease or other adverse condition in a population that can be attributed to a particular risk factor.

The PAF of ASDR, incidence and deaths of each cancer due to high BMI was employed to estimate the burden of high BMI, broken down by country, age group, sex, and year. In GBD 2019, the Theoretical Minimum Risk Exposure Level (TMREL) was used to determine the level of exposure to high BMI that would minimize the risk of DALYs and deaths of each type of cancer. This was done by measuring the estimated relative risk (RR) of DALYs and deaths of each cancer due to high BMI. The methods used to estimate the disease burdens, and the impact of risk factors have been extensively described in previous publications [28–22].

The following formula was used to calculate the PAF:

$$ PAF= \frac{{\int }_{i=n}^{m}RR\left(X\right)P\left(X\right)d\left(X\right)-RR\left(TMREL\right)}{{\int }_{i=n}^{m}RR\left(X\right)P\left(X\right)d\left(x\right)}$$

n is the lowest level of exposure observed, m is the highest level of exposure recorded, RR(x) is the relative risk at an exposure level of x and P(x) is the fraction of risk exposure.

We used the GBD estimated 95% uncertainty limits (UIs) for PAFs, and their trends or changes from 1990 to 2019 and analyzed mortality and DALYs descriptively by gender, country, cancer types, year, and then plotted the temporal trends of annual percentage change measures from 1990 to 2019 calculated by GBDs 2019. Finally, Spearman’s correlation test was used to examine the relationship between SDI and the ASMR of cancer attributable to high BMI in 2019 by location and year. Significance level was set at a p-value of ≤ 0.05 and 95% CI. Collected data were entered and analyzed with *SAS 9.4.* Detailed methods of data on high BMI, and calculation of mortality and DALYs have been reported in previous studies by GBD collaborators [22].

## Results

### Deaths and DALYs of Cancer Attributable to High BMI from 1990 to 2019 for both Sexes in GCC Countries

**Fig. 1:**
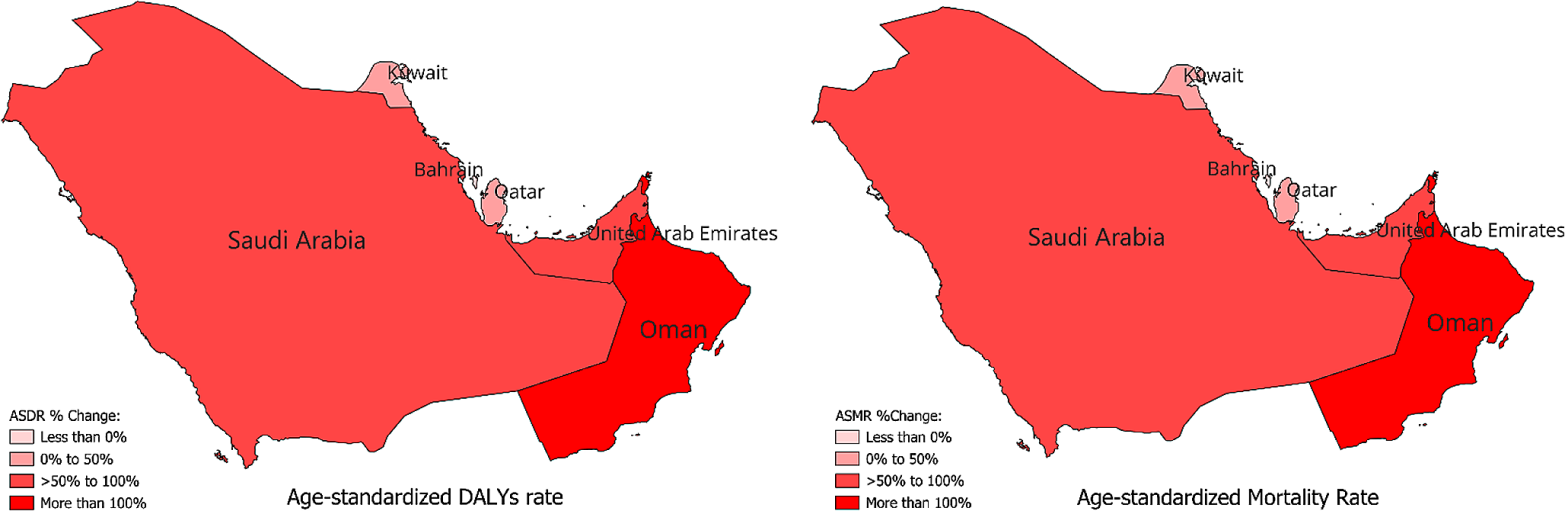
Percentage change in age-standardized DALYs and mortality rate in both sexes for all cancers attributable to high BMI From 1990 to 2019. ASDR, age-standardized DALYs rate; ASMR, age-standardized mortality rate; DALYs, disability-adjusted life-years; BMI, body mass index; UI, uncertainty interval

Between 1990 and 2019, all six GCC countries showed an increased number of overall cancer-related deaths increase (range between 398.73% in Bahrain to 1404.25% in United Arab Emirates), and DALYs (range 347.38% in Kuwait, to 1479.35% in the United Arab Emirates) reflecting significant increasing mortality burden cancer attributed to high BMI (Table 1). Overall, while each country showed different patterns and trajectories in disease burden and mortality rates, there were consistent disparities between sexes, with males generally experiencing higher rates of disease burden and mortality compared to females. These gender disparities were evident across various health indicators, including DALYs, ASDR, and the number of deaths. The GCC country’s cancer-related deaths attributable to high BMI have increased from 174.96 in 1990 to 1520.99 in 2019 for males and have increased from 134.19 in 1990 to 689.4 in 2019 for females (Table 1). Among the countries studied, United Arab Emirates showed the highest increase in the number of DALYs, with an overall percentage increase of 1479.35, driven primarily by a substantial increase in DALYs among males (1789.8%). Oman demonstrated the highest increase in the ASDR per 100,000 population, with a remarkable 152.16% percentage increase overall and a significant increase among males (178.77%) (Table 1; Fig. 1). Regarding overall changes in mortality rates, the United Arab Emirates, and Qatar showed the highest percentage change in the number of deaths, with a remarkable increase of 1404.25%, and 813.82% respectively. This increase was predominantly observed among males (United Arab Emirates 1762.24%, Qatar 935.31%), highlighting a concerning trend in mortality rates in the region. The highest increase in ASMR was in Oman with 186.25% percentage increase largely driven by the increase in ASMR among males with 225.24% percentage increase (Table 1).

**Table 1:** DALYs and Deaths of cancer attributable to high BMI in 2019 and percentage change from 1990 to 2019 both sexes and GCC countries

Countries	Sex	Number of DALYs			ASDR per (100,000)			Number of Deaths			ASMR per (100,000)		
		1990 (95% UI)	2019 (95% UI)	% change	1990 (95% UI)	2019 (95% UI)	% change	1990 (95% UI)	2019 (95% UI)	% change	1990 (95% UI)	2019 (95% UI)	% change
Saudi Arabia	Overall	5632.89 (3006.99–9141.68)	36554.20 (22768.17–54318.65)	548.94	85.77 (46.41–137.16)	169.13 (107.53–243.03)	97.19	199.63 (108.27–320.43)	1176.87 (740.84–1722.77)	489.53	3.57 (1.93–5.69)	7.14 (4.48–10.39)	100
	Female	2337.12 (1199.00–3972.12)	12532.44 (7416.31–19058.12)	436.23	94.37 (50.47–155.90)	173.35 (108.99–254.16)	83.69	90.15 (48.07–149.69)	448.87 (279.58–666.01)	397.91	4.03 (2.18–6.57)	7.56 (4.72–10.89)	87.59
	Male	3295.77 (1593.16–5726.79)	24021.76 (14508.66–36510.15)	628.87	78.91 (37.98–136.36)	164.98 (101.70–246.39)	109.07	109.47 (53.32–189.11)	728.00 (445.56–1094.34)	565.02	3.20 (1.53–5.49)	6.82 (4.13–10.20)	113.13
Bahrain	Overall	354.31 (209.61–522.81)	1769.26 (1104.46–2550.85)	399.35	181.29 (107.58 -265.71)	164.69 (102.34–236.12)	-9.16	12.63 (7.46–18.53)	62.99 (39.48–90.30)	398.73	8.09 (4.72–11.93)	8.04 (4.90–11.59)	-0.62
	Female	152.96 (88.56–234.47)	612.49 (354.33–940.56)	300.42	189.52 (112.55–282.75)	171.65 (104.57–253.50)	-9.43	5.92 (3.51–8.85)	25.19 (15.28–37.37)	325.51	8.49 (5.10–12.52)	8.54 (5.24–12.33)	0.59
	Male	201.35 (108.77–312.62)	1156.77 (685.57–1741.90)	474.51	170.07 (90.77–265.46)	158.03 (89.76–241.78)	-7.08	6.72 (3.63–10.44)	37.80 (22.00–57.28)	462.5	7.73 (4.05–12.28)	7.69 (4.27–11.98)	-0.52
Oman	Overall	482.90 (238.06–811.19)	3157.86 (1930.38–4669.62)	553.94	61.06 (29.77–102.43)	153.97 (94.64–221.94)	152.16	15.99 (7.77–26.94)	106.28 (64.92–155.08)	564.67	2.40 (1.15–4.11)	6.87 (4.22–9.98)	186.25
	Female	211.80 (103.33- 355.42)	1097.88 (652.96–1620.39)	418.36	67.92 (33.63–113.16)	155.59 (94.99–227.35)	129.08	7.82 (3.86–13.18)	43.04 (26.09–62.78)	450.38	2.73 (1.34–4.68)	7.04 (4.29–10.34)	157.88
	Male	271.11 (117.90–497.48)	2059.98 (1165.45–3160.39)	659.83	53.66 (22.08–99.80)	149.59 (87.05–227.10)	178.77	8.17 (3.43–15.13)	63.25 (36.39–97.59)	674.17	2.06 (0.83–3.84)	6.70 (3.88–10.21)	225.24
Kuwait	Overall	865.11 (540.73–1221.59)	3870.31 (2513.61–5316.24)	347.38	122.11 (76.07–173.01)	141.68 (90.93–198.33)	16.03	29.36 (18.16–41.66)	147.58 (94.52–206.70)	402.66	5.20 (3.16–7.47)	6.54 (4.12–9.24)	25.77
	Female	352.18 (220.14–504.13)	1365.25 (852.43–1984.59)	287.66	163.47 (104.75–228.81)	141.74 (91.96–197.32)	-13.29	13.90 (8.82–19.65)	56.48 (36.12–79.03)	306.33	7.09 (4.48–9.95)	6.65 (4.31–9.37)	-6.21
	Male	512.93 (291.36–747.12)	2505.06 (1517.81–3699.69)	388.38	97.21 (53.06–144.20)	143.15 (84.37–213.88)	47.26	15.46 (8.54–22.74)	91.10 (53.74–135.46)	489.26	3.92 (2.11–5.94)	6.49 (3.72–9.70)	65.56
Qatar	Overall	292.69 (169.12–447.33)	2699.02 (1679.82–4005.31)	822.14	242.93 (140.13–367.19)	291.55 (181.53–423.70)	20.01	9.84 (5.71–14.90)	89.92 (56.16–132.52)	813.82	11.40 (6.52–17.15)	16.13 (9.96–23.42)	41.49
	Female	77.09 (40.96–124.83)	504.45 (260.98–818.92)	554.37	265.87 (154.16–400.07)	350.36 (217.05–502.53)	31.78	3.26 (1.84–5.05)	21.90 (12.97–32.97)	571.78	12.33 (7.01–18.56)	19.31 (12.13–27.45)	56.61
	Male	215.60 (113.84–344.95)	2194.57 (1325.59–3358.35)	917.89	232.48 (118.74- 378.07)	273.46 (165.08–412.31)	17.63	6.57 (3.46–10.55)	68.02 (40.71–104.10)	935.31	11.05 (5.49–18.23)	15.14 (8.98–22.66)	37.01
UAE	Overall	1452.15 (765.45–2427.15)	22934.50 (11940.71–37486.81)	1479.35	235.58 (124.23–375.20)	358.50 (197.21–561.46)	52.18	41.65 (21.63–68.46)	626.52 (330.92–1004.13)	1404.25	9.89 (5.12–15.72)	15.24 (8.54–23.31)	54.1
	Female	386.22 (175.78–683.92)	2790.54 (1263.58–4756.77)	622.53	254.16 (129.93–418.69)	279.29 (160.03–433.61)	9.89	13.05 (6.27–22.30)	93.92 (49.44–152.25)	619.69	10.59 (5.41–17.39)	11.96 (6.85–18.49)	12.94
	Male	1065.93 (519.73–1795.87)	20143.96 (10389.16–33539.93)	1789.8	224.26 (107.14–380.51)	386.44 (206.92–630.43)	72.32	28.60 (13.95–48.06)	532.60 (277.64–888.99)	1762.24	9.51 (4.72–16.07)	16.52 (8.88–26.48)	73.71

BMI, body mass index; GCC, gulf cooperation council; ASDR, age-standardized DALYs rate; ASMR, age-standardized mortality rate; DALYs, disability-adjusted life-years; UAE, United Arab of Emirates; UI, uncertainty interval

### Percentage Change from 1990 to 2019 in DALYs, and Death of Cancer Attributable to High BMI by GCC Countries, Both Sexes, and Cancer Types

In Table 2, the highest increases in the number of deaths and DALYs for both sexes across all countries were observed for pancreatic cancer (ranged between 6 and 33 times higher than baseline), kidney cancer (ranged between 6 and 12 times higher than baseline), ovarian cancer (ranged between 3 and 17 times higher than baseline), and colon and rectum cancer (ranged between 3 and 12 times higher than baseline). When adjusting for age, pancreatic cancer, kidney cancer, and colon and rectum cancer exhibited the highest increases in ASMR and ASDR among all included countries (Table 2). Notably, the most substantial percentage increase occurred in pancreatic cancer in the United Arab Emirates, with a remarkable surge of 3261%, which translates to approximately 33.61 times higher than the baseline (Table 2).

**Table 2:** Burden of DALYs and ASDR of cancer attributable to high BMI and percentage change from 1990 to 2019 by GCC countries, both sex, and cancer types

Countries	Cancer types	Number of DALYs			Number of Deaths			ASDR per 100,00			ASMR per 100,00		
		1990 95% UI	2019 95% UI	% change	1990 95% UI	2019 95% UI	% change	1990 95% UI	2019 95% UI	% change	1990 95% UI	2019 95% UI	% change
Saudi Arabia	*Breast cancer*	-315.76 (-864.18, 46.32)	-3532.27 (-8304.16, -290.43)	-1019	-0.14 (-13.14, 10.79)	-26.95 (-134.37, 51.22)	-18,897	1.17 (-4.31, 6.27)	3.77 (0.37, 14.66)	222.5	0.15 (-0.02, 0.37)	0.38 (0, 0.8)	155.4
	*Colon and rectum cancer*	953.82 (456.84, 1689.95)	9444.86 (5461.09, 14123.25)	890.2	32.05 (15.31, 57.31)	281.43 (163.95, 416.8)	778.1	13.54 (6.49, 24.24)	38.11 (0.99, 56.51)	181.4	0.55 (0.26, 0.99)	1.56 (0.92, 2.31)	182.7
	*Esophageal cancer*	724.55 (224.45, 1354.05)	3646.36 (1341.21, 6257.28)	403.3	26.05 (8.01, 48.39)	117.17 (42.66, 197.34)	349.8	11.1 (3.46, 20.57)	16.06 (0.87, 26.89)	44.62	0.47 (0.14, 0.87)	0.69 (0.25, 1.14)	48.86
	*Gallbladder and biliary tract cancer*	442.92 (212.93, 889.24)	2249.82 (1241.74, 3648.75)	408	16.99 (8.12, 33.94)	75.28 (42.3, 124.02)	343	7.1 (3.38, 14.34)	10.54 (0.65, 17.24)	48.49	0.33 (0.15, 0.65)	0.48 (0.27, 0.78)	47.83
	*Kidney cancer*	236.67 (116.51, 398.29)	3114.53 (1787.82, 4881.27)	1216	7.68 (3.77, 13.03)	89.69 (51.47, 138.08)	1068	3.31 (1.63, 5.6)	12.26 (0.22, 18.68)	270.7	0.13 (0.06, 0.22)	0.47 (0.28, 0.71)	267.7
	*Leukemia*	756.34 (339.06, 1357.96)	4142.72 (2208.19, 6848.16)	447.7	20.79 (9.53, 37.51)	103.88 (55.39, 170.57)	399.6	8.51 (3.91, 15.31)	12.96 (0.55, 20.96)	52.25	0.3 (0.14, 0.55)	0.45 (0.24, 0.73)	50.05
	*Liver cancer*	1472.65 (562.35, 2844.13)	5753.65 (2565.65, 10001.37)	290.7	50.99 (19.6, 97.74)	196.5 (88.08, 340.7)	285.4	21.87 (8.41, 42.12)	28.23 (1.68, 48.77)	29.08	0.88 (0.34, 1.68)	1.23 (0.55, 2.12)	40.96
	*Multiple myeloma*	104.85 (42.94, 215.31)	721.42 (327.32, 1311.24)	588	3.69 (1.5, 7.75)	22.48 (9.93, 40.03)	509.9	1.59 (0.65, 3.36)	3.12 (0.14, 5.54)	95.47	0.06 (0.03, 0.14)	0.13 (0.06, 0.23)	95.27
	*Non-Hodgkin lymphoma*	536.85 (186.92, 1061.03)	3511.91 (1572.9, 6054.3)	554.2	17.08 (5.94, 34.27)	98.2 (43.01, 166.87)	475	7.05 (2.48, 14.21)	13.28 (0.57, 22.61)	88.38	0.28 (0.1, 0.57)	0.52 (0.23, 0.88)	83.79
	*Ovarian cancer*	71.94 (-2.16, 175.99)	867.63 (-16.59, 2111.96)	1106	2.14 (-0.07, 5.26)	23.66 (-0.45, 56.92)	1003	0.93 (-0.03, 2.25)	2.97 (0.08, 7.17)	220.8	0.03 (0, 0.08)	0.1 (0, 0.24)	216.3
	*Pancreatic cancer*	172.04 (52.36, 354.07)	3141.49 (1159.88, 5787.4)	1726	6.17 (1.9, 12.82)	100 (36.87, 184.39)	1521	2.63 (0.81, 5.48)	13.52 (0.23, 24.94)	414.6	0.11 (0.03, 0.23)	0.57 (0.22, 1.05)	422.7
	*Thyroid cancer*	118.46 (54.35, 210.82)	1244.21 (647.62, 2014.02)	950.3	3.6 (1.68, 6.54)	28.67 (14.45, 45.57)	696.5	1.55 (0.71, 2.83)	4.36 (0.12, 6.82)	181.3	0.06 (0.03, 0.12)	0.15 (0.07, 0.23)	143.8
	*Uterine cancer*	357.56 (195.89, 592.78)	2247.89 (1413.03, 3197.2)	528.7	12.53 (7.01, 20.51)	66.87 (42.12, 92.21)	433.6	5.43 (3.03, 8.92)	9.96 (0.37, 14.1)	83.51	0.23 (0.13, 0.37)	0.4 (0.23, 0.56)	75.41
Bahrain	*Breast cancer*	-2.27 (-41.02, 38.21)	-48.63 (-257.08, 115.31)	-2043	0.68 (-0.45, 2)	2.82 (-2.93, 8.66)	314	14.5 (-2.32, 34.24)	11.39 (1.7, 26.82)	-21.46	0.83 (0.17, 1.7)	0.86 (0.23, 1.65)	3.51
	*Colon and rectum cancer*	69.05 (37.66, 105.91)	415.17 (232.82, 636.31)	501.2	2.3 (1.25, 3.53)	13.69 (7.69, 20.95)	496.7	32.03 (17.36, 49.37)	34.93 (2.18, 53.59)	9.04	1.39 (0.74, 2.18)	1.66 (0.9, 2.56)	19.38
	*Esophageal cancer*	54.07 (17.56, 97.26)	164.28 (53.21, 277.12)	203.9	1.99 (0.65, 3.6)	5.82 (1.9, 9.8)	192.5	28.4 (9.29, 52.03)	14.51 (2.39, 24.84)	-48.91	1.27 (0.41, 2.39)	0.71 (0.24, 1.23)	-44.24
	*Gallbladder and biliary tract cancer*	15.01 (8.14, 23.85)	59.67 (31.2, 94.94)	297.6	0.57 (0.31, 0.9)	2.23 (1.19, 3.55)	293	8.2 (4.5, 13.1)	5.89 (0.62, 9.32)	-28.25	0.39 (0.21, 0.62)	0.31 (0.17, 0.48)	-21.84
	*Kidney cancer*	25.87 (14.79, 39.77)	168.63 (96.34, 250.09)	551.8	0.87 (0.49, 1.34)	5.41 (3.08, 8.15)	525.4	12.14 (6.85, 18.88)	13.37 (0.79, 20.1)	10.2	0.51 (0.28, 0.79)	0.59 (0.34, 0.9)	16.75
	*Leukemia*	50.11 (25.15, 80.37)	177.1 (90.88, 285.68)	253.5	1.36 (0.69, 2.22)	5.17 (2.67, 8.2)	278.8	17.42 (8.77, 28.5)	13.87 (1.14, 21.92)	-20.41	0.7 (0.34, 1.14)	0.6 (0.31, 0.95)	-14.57
	*Liver cancer*	49.4 (21.09, 87.18)	274.01 (121.04, 490.07)	454.7	1.75 (0.74, 3.13)	9.54 (4.27, 16.84)	445.8	24.44 (10.28, 44.17)	23.66 (2.01, 41.75)	-3.18	1.1 (0.47, 2.01)	1.14 (0.5, 1.99)	3.56
	*Multiple myeloma*	9.34 (3.9, 17.01)	46.57 (19.44, 89.71)	398.7	0.34 (0.14, 0.62)	1.6 (0.66, 2.99)	377.1	4.79 (1.97, 8.97)	3.96 (0.39, 7.42)	-17.16	0.21 (0.08, 0.39)	0.18 (0.08, 0.33)	-12.46
	*Non-Hodgkin lymphoma*	19.5 (8.3, 34.33)	114.35 (48.89, 196.7)	486.5	0.57 (0.25, 1)	3.42 (1.47, 5.87)	498.2	7.42 (3.14, 12.92)	8.97 (0.54, 15.21)	20.9	0.3 (0.13, 0.54)	0.39 (0.17, 0.66)	27.01
	*Ovarian cancer*	8.4 (-0.31, 19.4)	54.02 (-1.22, 133.51)	542.7	0.26 (-0.01, 0.62)	1.69 (-0.04, 4.13)	540.3	3.69 (-0.14, 8.62)	4.05 (0.36, 9.62)	9.85	0.15 (-0.01, 0.36)	0.18 (0, 0.41)	18.12
	*Pancreatic cancer*	22.81 (8.01, 42.22)	168.87 (53.72, 322.31)	640.2	0.82 (0.29, 1.51)	5.98 (1.93, 11.28)	628	11.62 (4.11, 21.46)	14.9 (0.97, 28.03)	28.13	0.52 (0.19, 0.97)	0.74 (0.25, 1.37)	40.17
	*Thyroid cancer*	5.25 (2.68, 8.6)	29.7 (15.71, 48.11)	465.4	0.18 (0.09, 0.3)	0.95 (0.5, 1.51)	420.1	2.7 (1.41, 4.43)	2.83 (0.2, 4.51)	4.95	0.12 (0.06, 0.2)	0.14 (0.07, 0.22)	9.95
	*Uterine cancer*	27.77 (17.79, 40.56)	145.53 (86.47, 205.86)	424	0.95 (0.6, 1.4)	4.67 (2.82, 6.48)	392.1	13.94 (8.82, 20.67)	12.37 (0.89, 17.13)	-11.28	0.59 (0.37, 0.89)	0.56 (0.36, 0.76)	-5.81
Oman	*Breast cancer*	2.3 (-32.01, 36.72)	-2.62 (-220.55, 215.18)	-213.9	0.65 (-0.27, 1.94)	5.05 (-1.68, 12.97)	672.4	3.41 (-0.43, 9.02)	12.2 (0.4, 25.73)	258	0.17 (0.02, 0.4)	0.64 (0.21, 1.23)	266.4
	*Colon and rectum cancer*	77.79 (34.69, 144.61)	581.44 (326.23, 963.75)	647.4	2.48 (1.09, 4.69)	19.28 (10.8, 30.83)	678.9	9.32 (4.09, 17.71)	27.77 (0.71, 44.71)	198.1	0.36 (0.15, 0.71)	1.3 (0.72, 1.99)	257.3
	*Esophageal cancer*	74.25 (22.53, 145.78)	363.49 (124.56, 656.76)	389.6	2.51 (0.73, 5.05)	12.68 (4.34, 22.13)	404.8	9.57 (2.8, 19.29)	18.13 (0.78, 31.48)	89.45	0.38 (0.1, 0.78)	0.82 (0.3, 1.41)	118.9
	*Gallbladder and biliary tract cancer*	30.17 (13.84, 53.75)	107.68 (59.8, 168.52)	256.9	1.12 (0.51, 2)	4.12 (2.32, 6.51)	268.7	4.27 (1.93, 7.62)	6.19 (0.34, 9.7)	44.93	0.19 (0.08, 0.34)	0.31 (0.18, 0.49)	66.44
	*Kidney cancer*	18.9 (8.45, 33.36)	201.34 (119.13, 303.23)	965.4	0.59 (0.26, 1.05)	6.26 (3.74, 9.31)	956	2.25 (0.99, 4.01)	8.85 (0.15, 13.11)	293.2	0.08 (0.04, 0.15)	0.38 (0.22, 0.55)	343.9
	*Leukemia*	66.38 (29.59, 120.59)	344.07 (181.24, 566.29)	418.3	1.84 (0.82, 3.39)	9.25 (4.9, 14.94)	402.9	6.62 (2.92, 12.2)	11.82 (0.45, 18.93)	78.63	0.24 (0.1, 0.45)	0.49 (0.26, 0.79)	104.8
	*Liver cancer*	99.68 (35.09, 212.66)	678.04 (287.06, 1210.07)	580.2	3.13 (1.08, 6.69)	20.93 (8.88, 36.81)	569.2	11.69 (4, 25.25)	27.64 (0.92, 48.56)	136.5	0.43 (0.14, 0.92)	1.1 (0.5, 1.95)	158.6
	*Multiple myeloma*	12.16 (4.8, 25.93)	83.23 (34.68, 154.27)	584.2	0.42 (0.16, 0.9)	2.86 (1.22, 5.15)	585.7	1.6 (0.62, 3.43)	4.16 (0.13, 7.44)	159.5	0.06 (0.02, 0.13)	0.18 (0.08, 0.33)	187.8
	*Non-Hodgkin lymphoma*	48.99 (17.28, 100.77)	329.77 (140.65, 604.93)	573.2	1.49 (0.52, 3.06)	9.94 (4.38, 17.84)	565.1	5.49 (1.9, 11.28)	13.63 (0.43, 24.14)	148.2	0.21 (0.07, 0.43)	0.58 (0.26, 1.03)	181.8
	*Ovarian cancer*	5.23 (-0.12, 13.84)	65.38 (-1.39, 155.14)	1150	0.16 (0, 0.43)	2.03 (-0.05, 4.84)	1148	0.62 (-0.01, 1.63)	2.78 (0.06, 6.56)	349.9	0.02 (0, 0.06)	0.11 (0, 0.26)	386.5
	*Pancreatic cancer*	15.47 (4.64, 32.99)	248.66 (87.17, 467.31)	1508	0.54 (0.16, 1.14)	8.96 (3.26, 16.37)	1571	2.04 (0.62, 4.35)	12.93 (0.18, 23.48)	535.5	0.08 (0.03, 0.18)	0.61 (0.23, 1.09)	630.3
	*Thyroid cancer*	7.83 (3.63, 14.14)	56.6 (28.17, 94.14)	622.7	0.23 (0.1, 0.42)	1.48 (0.78, 2.4)	552.7	0.89 (0.4, 1.65)	2.31 (0.06, 3.72)	159.6	0.03 (0.01, 0.06)	0.09 (0.05, 0.15)	181.2
	*Uterine cancer*	23.75 (12.94, 38.84)	100.77 (65.08, 139.42)	324.3	0.83 (0.44, 1.36)	3.45 (2.2, 4.75)	313.6	3.3 (1.72, 5.4)	5.55 (0.22, 7.7)	68.27	0.14 (0.07, 0.22)	0.25 (0.15, 0.34)	81
Kuwait	*Breast cancer*	-62.74 (-154.45, 9.79)	-165.75 (-588.33, 153.26)	-164.2	0.26 (-2.1, 2.46)	3.79 (-7.14, 13.91)	1377	6.22 (-3.39, 16.45)	7.46 (0.89, 17.78)	19.9	0.44 (0.06, 0.89)	0.48 (0.12, 0.93)	8.67
	*Colon and rectum cancer*	157.08 (91.9, 229.57)	1080.22 (652.2, 1571.85)	587.7	5.05 (2.91, 7.5)	40.6 (23.91, 59.76)	703.8	20.27 (11.58, 30.25)	37.78 (1.28, 55.6)	86.38	0.85 (0.46, 1.28)	1.79 (1.02, 2.67)	111.3
	*Esophageal cancer*	98.09 (34.72, 160.3)	334.22 (127.84, 545.97)	240.7	3.35 (1.21, 5.58)	13.2 (4.84, 21.74)	293.9	13.53 (4.89, 22.49)	11.64 (0.99, 19.15)	-13.98	0.58 (0.2, 0.99)	0.58 (0.21, 0.97)	-0.34
	*Gallbladder and biliary tract cancer*	72.62 (41.18, 112.61)	177.47 (102.1, 269.09)	144.4	2.65 (1.51, 4.15)	6.9 (3.92, 10.53)	159.8	10.95 (6.15, 17.13)	6.34 (0.79, 9.73)	-42.09	0.5 (0.28, 0.79)	0.3 (0.17, 0.47)	-39.49
	*Kidney cancer*	58.96 (35.93, 85.76)	339.75 (207.49, 499.63)	476.2	1.81 (1.09, 2.67)	11.33 (6.78, 16.67)	524.2	7.33 (4.37, 10.88)	10.64 (0.43, 15.82)	45.21	0.29 (0.17, 0.43)	0.45 (0.26, 0.66)	54.6
	*Leukemia*	156.26 (83.07, 244.33)	403.32 (229.9, 617.45)	158.1	4.04 (2.13, 6.31)	12.08 (6.74, 18.75)	198.9	14.47 (7.66, 22.85)	11 (0.85, 16.98)	-23.98	0.53 (0.27, 0.85)	0.44 (0.24, 0.69)	-17.4
	*Liver cancer*	116.86 (50.69, 199.29)	400.79 (187.42, 693.37)	243	3.69 (1.61, 6.34)	14.53 (6.73, 25.11)	293.7	14.7 (6.35, 25.46)	13.68 (0.99, 23.71)	-6.91	0.57 (0.25, 0.99)	0.61 (0.28, 1.06)	6.28
	*Multiple myeloma*	17.95 (8.24, 31.04)	90.17 (42.68, 158.67)	402.5	0.59 (0.27, 1.02)	3.07 (1.46, 5.39)	419.8	2.45 (1.11, 4.26)	2.91 (0.17, 5.12)	18.99	0.1 (0.04, 0.17)	0.12 (0.06, 0.21)	23.17
	*Non-Hodgkin lymphoma*	82.65 (36.67, 141.26)	295.58 (133.25, 499.67)	257.7	2.34 (1.04, 4.04)	9.61 (4.33, 16.06)	311.1	8.85 (3.98, 15.19)	9.01 (0.58, 15.09)	1.77	0.34 (0.15, 0.58)	0.38 (0.17, 0.64)	12.38
	*Ovarian cancer*	21.09 (-0.51, 48.49)	95.76 (-2.32, 224.3)	354	0.62 (-0.02, 1.42)	2.9 (-0.07, 6.76)	367.8	2.42 (-0.06, 5.53)	2.67 (0.21, 6.17)	10.03	0.09 (0, 0.21)	0.1 (0, 0.23)	9.75
	*Pancreatic cancer*	54.94 (19.71, 103.59)	354.95 (115.67, 665.84)	546.1	1.94 (0.7, 3.7)	13.85 (4.46, 26.09)	612.1	7.94 (2.86, 15)	12.66 (0.65, 23.78)	59.5	0.35 (0.13, 0.65)	0.61 (0.19, 1.17)	73.38
	*Thyroid cancer*	22.52 (12.43, 34.5)	90.01 (47.6, 142.3)	299.7	0.65 (0.35, 1.01)	2.86 (1.5, 4.51)	339	2.85 (1.54, 4.39)	3.01 (0.18, 4.74)	5.6	0.11 (0.06, 0.18)	0.13 (0.07, 0.2)	12.32
	*Uterine cancer*	68.84 (49.14, 90.2)	373.8 (262.67, 504.44)	443	2.36 (1.67, 3.12)	12.86 (8.92, 17.36)	445.3	10.13 (7.19, 13.4)	12.88 (0.6, 17.44)	27.14	0.45 (0.31, 0.6)	0.56 (0.39, 0.76)	25.09
Qatar	*Breast cancer*	-24.52 (-56.22, 0.16)	-161.22 (-413.23, 22.67)	-557.4	-0.13 (-0.91, 0.55)	0.24 (-5.53, 5.2)	-289.7	13.65 (-0.74, 31.09)	15.58 (1.75, 32.12)	14.14	0.85 (0.21, 1.75)	0.99 (0.32, 1.79)	16.03
	*Colon and rectum cancer*	47.12 (25.16, 74.23)	535.42 (312.96, 813.27)	1036	1.46 (0.78, 2.3)	16.63 (9.76, 25.08)	1041	33.08 (17.74, 52.63)	54.4 (2.47, 81.13)	64.46	1.54 (0.81, 2.47)	3.09 (1.83, 4.59)	100.6
	*Esophageal cancer*	39.89 (14.49, 69.53)	282.6 (92.09, 528.15)	608.4	1.31 (0.46, 2.24)	9.33 (3.14, 16.94)	611.4	31.1 (11, 53.36)	32.19 (2.58, 57.51)	3.52	1.51 (0.51, 2.58)	2 (0.67, 3.56)	32.86
	*Gallbladder and biliary tract cancer*	16.54 (8.82, 25.91)	83.12 (39.9, 137.25)	402.6	0.59 (0.32, 0.92)	2.92 (1.47, 4.75)	395.5	15.37 (7.98, 24.12)	10.17 (1.26, 15.92)	-33.8	0.8 (0.41, 1.26)	0.59 (0.31, 0.91)	-27.14
	*Kidney cancer*	20.63 (11.25, 33.22)	239.74 (135.02, 375.17)	1062	0.65 (0.35, 1.05)	7.56 (4.16, 11.94)	1066	15.08 (7.81, 24.37)	23.67 (1.08, 36.77)	56.98	0.66 (0.34, 1.08)	1.29 (0.69, 2.01)	94.76
	*Leukemia*	39.97 (19.86, 66.3)	303.95 (156.95, 523.24)	660.4	1.01 (0.51, 1.61)	7.61 (4.06, 12.67)	656.5	18.22 (9.5, 28.96)	20.52 (1.27, 32.9)	12.62	0.78 (0.39, 1.27)	1.12 (0.58, 1.91)	43.74
	*Liver cancer*	98.49 (42.17, 180.21)	834.67 (366.41, 1506.36)	747.5	3.29 (1.39, 5.95)	28.15 (12.48, 49.89)	756	78.67 (33.15, 141.75)	83.63 (6.34, 144.83)	6.3	3.56 (1.5, 6.34)	4.46 (1.97, 7.66)	25.2
	*Multiple myeloma*	4.47 (1.86, 8.36)	47.24 (17.99, 92.8)	957.5	0.14 (0.06, 0.26)	1.5 (0.57, 2.91)	965.2	3.26 (1.28, 6.07)	4.31 (0.26, 8.51)	32.32	0.14 (0.05, 0.26)	0.21 (0.08, 0.42)	49.98
	*Non-Hodgkin lymphoma*	21.25 (9.32, 38.08)	196.98 (88.85, 344.75)	826.8	0.59 (0.26, 1.04)	5.32 (2.43, 9.23)	804.8	11.72 (5.19, 20.74)	14.69 (0.89, 25.17)	25.35	0.5 (0.22, 0.89)	0.74 (0.33, 1.27)	47.99
	*Ovarian cancer*	3.64 (-0.14, 8.81)	47.79 (-0.97, 108)	1212	0.11 (0, 0.26)	1.42 (-0.03, 3.23)	1188	2.41 (-0.08, 5.81)	3.38 (0.25, 7.81)	40.25	0.1 (0, 0.25)	0.14 (0, 0.33)	39.9
	*Pancreatic cancer*	11.68 (3.2, 23.14)	182.93 (52.56, 365.08)	1466	0.39 (0.11, 0.77)	6.06 (1.75, 12.02)	1467	9.11 (2.47, 17.79)	17.82 (0.83, 33.91)	95.61	0.42 (0.12, 0.83)	0.96 (0.3, 1.83)	126
	*Thyroid cancer*	4.03 (1.98, 6.95)	36.61 (17.28, 63.4)	808.2	0.11 (0.06, 0.19)	0.9 (0.43, 1.54)	685.3	2.62 (1.33, 4.43)	2.93 (0.2, 4.94)	11.83	0.12 (0.06, 0.2)	0.14 (0.07, 0.24)	20.4
	*Uterine cancer*	9.49 (5.42, 13.85)	69.19 (31.27, 101.38)	629.2	0.33 (0.17, 0.48)	2.28 (0.93, 3.34)	598.8	8.65 (4.09, 13.32)	8.26 (0.66, 12.07)	-4.49	0.41 (0.19, 0.66)	0.41 (0.14, 0.6)	-1.01
United Arab Emirates	*Breast cancer*	-44.5 (-136, 33.62)	-1207.79 (-2805.33, -55.7)	-2614	0.18 (-2.06, 2.59)	-13.97 (-49.73, 14.24)	-7782	13.87 (1.22, 31.76)	10.61 (1.4, 26.16)	-23.48	0.66 (0.15, 1.4)	0.61 (0.11, 1.23)	-8.54
	*Colon and rectum cancer*	244.07 (121.69, 433.68)	3414.28 (1929.24, 5205.36)	1299	7.25 (3.64, 12.64)	96.33 (55.19, 147.05)	1228	43.93 (21.91, 75.72)	66.18 (3.63, 102.38)	50.65	2.07 (1.01, 3.63)	3.36 (1.84, 5.31)	62.43
	*Esophageal cancer*	354.38 (68.86, 722.47)	5906.2 (1165.65, 11769.65)	1567	10.18 (2.08, 20.93)	160.39 (31.45, 321.68)	1476	55.21 (11.07, 115.32)	85.07 (4.85, 175.17)	54.1	2.27 (0.45, 4.85)	3.53 (0.7, 7.23)	55.5
	*Gallbladder and biliary tract cancer*	72.83 (31.24, 137.93)	671.76 (274.2, 1194.07)	822.3	2.28 (0.95, 4.33)	18.54 (7.69, 33.16)	713.8	14.1 (5.39, 27.97)	10.83 (1.29, 19.26)	-23.16	0.64 (0.24, 1.29)	0.48 (0.2, 0.86)	-25.41
	*Kidney cancer*	126.14 (43.61, 318.26)	3180.57 (1117.73, 5899.57)	2421	3.3 (1.09, 8.73)	81.28 (27.35, 149.82)	2361	16.01 (4.84, 43.1)	39.6 (1.66, 74.67)	147.3	0.61 (0.17, 1.66)	1.5 (0.44, 2.83)	147.5
	*Leukemia*	183.21 (92.67, 305.91)	1887.7 (1017.48, 3016.9)	930.3	4.62 (2.39, 7.5)	46.79 (25.95, 73.64)	912.4	22.4 (11.02, 37.6)	23.45 (1.55, 37.73)	4.7	0.88 (0.42, 1.55)	0.9 (0.48, 1.47)	2.61
	*Liver cancer*	157.54 (51.74, 347.4)	2510.19 (703.52, 6517.83)	1493	4.35 (1.4, 9.98)	67.46 (18.52, 177.83)	1451	22.3 (7.24, 54.49)	35.49 (2.24, 99.9)	59.13	0.9 (0.3, 2.24)	1.45 (0.38, 4.17)	61.78
	*Multiple myeloma*	31.19 (9.61, 69.6)	477.19 (142.13, 1213.97)	1430	0.87 (0.26, 1.96)	12.51 (3.82, 31.93)	1343	4.57 (1.36, 10.73)	6.38 (0.42, 16.81)	39.66	0.18 (0.05, 0.42)	0.25 (0.07, 0.65)	38.35
	*Non-Hodgkin lymphoma*	147.33 (44.85, 340.5)	1803.59 (640.37, 3598.47)	1124	3.47 (1.01, 8.11)	42.37 (14.36, 83.18)	1121	14 (3.66, 34.12)	17.95 (1.22, 35.98)	28.22	0.49 (0.12, 1.22)	0.6 (0.18, 1.22)	22.66
	*Ovarian cancer*	13.2 (-0.39, 32.78)	221.34 (-4.26, 580.59)	1576	0.35 (-0.01, 0.85)	5.61 (-0.11, 14.84)	1522	1.71 (-0.06, 4.46)	2.74 (0.17, 7.14)	60.27	0.06 (0, 0.17)	0.1 (0, 0.27)	59.31
	*Pancreatic cancer*	92.91 (18.02, 217.16)	3122.92 (378.27, 7455.58)	3261	2.71 (0.55, 6.41)	86.38 (10.89, 203.53)	3083	15.02 (3.14, 34.95)	47.46 (1.43, 106.65)	215.9	0.62 (0.13, 1.43)	1.98 (0.34, 4.33)	220
	*Thyroid cancer*	28.41 (10.66, 54.64)	540.3 (189.9, 1043.77)	1802	0.69 (0.26, 1.35)	12.2 (4.21, 23.38)	1661	3.46 (1.24, 6.69)	6.19 (0.27, 12.21)	79.01	0.13 (0.04, 0.27)	0.22 (0.07, 0.43)	66.46
	*Uterine cancer*	45.42 (18.44, 87.2)	406.26 (183.93, 674.24)	794.5	1.39 (0.53, 2.76)	10.63 (4.54, 18.28)	663.9	9.01 (3.1, 18.8)	6.54 (0.83, 11.93)	-27.34	0.39 (0.13, 0.83)	0.27 (0.1, 0.5)	-31.43

### Temporal Trends of Deaths, DALY’s ASDR and ASMR Between the Years 1990 to 2019

**Fig. 2:**
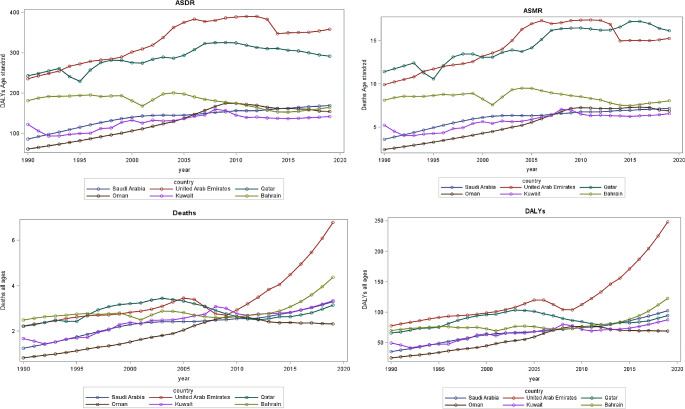
The temporal trends of the (**A**) deaths, (**B**) DALYs, (**C**) ASMR, and (**D**) ASDR for cancerattributable to high BMI from 1990 to 2019 overall. ASDR, age-standardizedDALYs rate; ASMR, age-standardized mortality rate; BMI, body mass index; DALYs,disability

In 2019, there were more than 2,210 thousand (2.16%) deaths can be attributed to high BMI, corresponding to an ASMR of 59.96 per 100,000. DALYs resulting from cancer were estimated to be 322,168 thousand, of which 1.28% (4152 thousand) was attributed to high BMI, with an ASDR of 133.93 per 100,000. The time trend showed a consistent upward trend of death numbers, DALYs, ASDR, and ASMR between the years 1990 and 2019 in all GCC countries except for Bahrain, which (Fig. 2; Supplementary Table 3 A, and B). The United Arab Emirates had a more than 14-folded increase in the death rate and a nearly 15-folded increase in DALYs. Whereas, Qatar had nearly 8-foldeed increase in death, and 8-folded increase of DALY’s of cancer attributable to high BMI between the 1990 to 2019 (Supplementary Table 3 A). The ASDR and ASMR of cancer attributable to high BMI trend increase (ranging between 30 and 90% increase) were the highest in the United Arab of Emirates and Qatar rate between the years 1990 to 2019 compared to other GCC countries. (Fig. 2; Supplementary Table 3B).

### DALYs and Deaths of Specific Cancer Types Attributable to High BMI in 2019 and Percentage Change from 1990 to 2019 by Cancer Types and GCC Countries (Female)

**Fig. 3:**
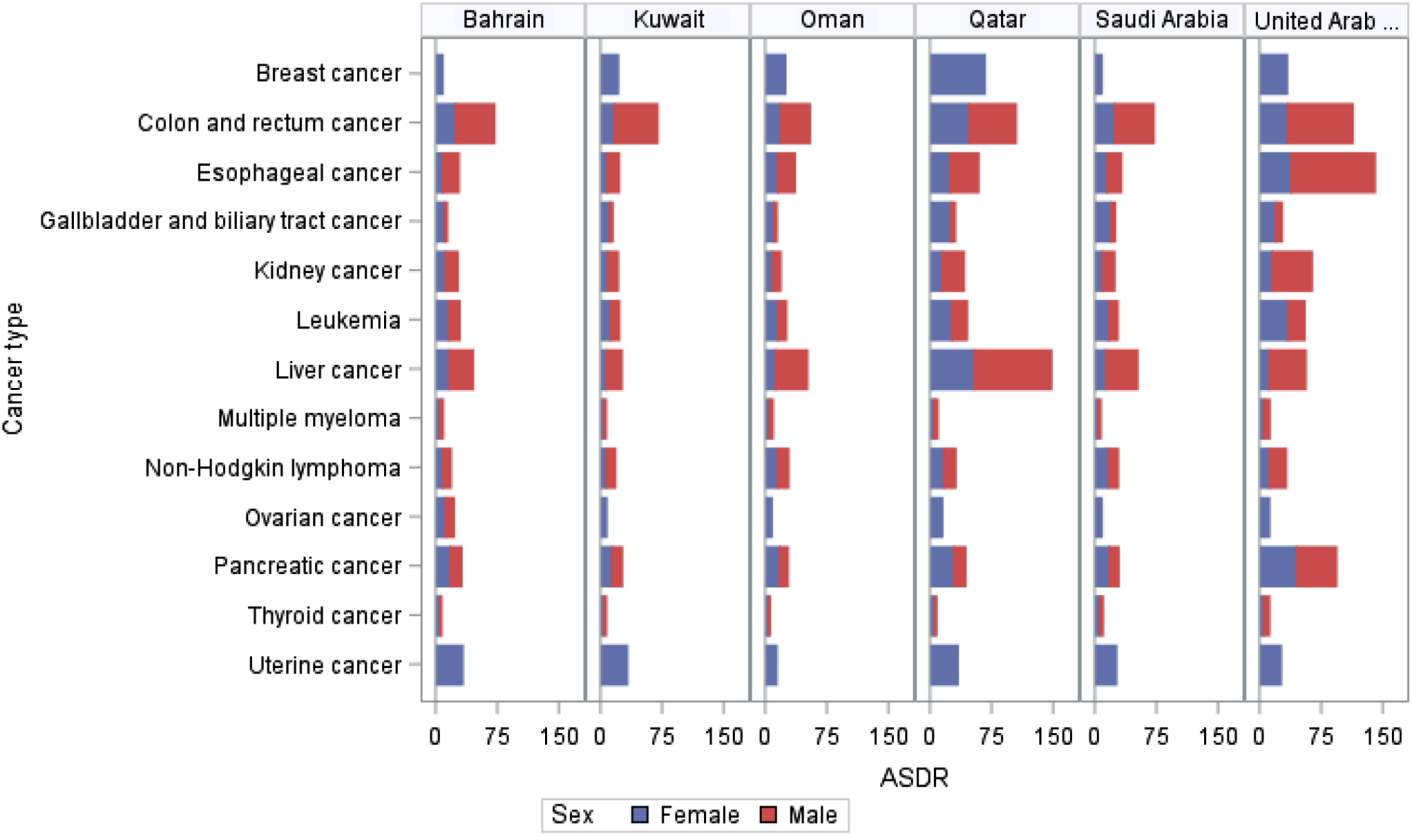
DALYs of specific type of cancer attributable to high BMI in 2019 across GCC countries

In GBD 2019, 13 cancer types were found to be affected by high BMI among females, including esophageal cancer, breast cancer, gallbladder and biliary tract cancer, liver cancer, uterine cancer, pancreatic cancer, multiple myeloma, colon and rectum cancer, thyroid cancer, kidney cancer, ovarian cancer, non-Hodgkin lymphoma, and leukemia. Breast cancer showed a consistent decrease in DALYs and deaths among females in the included countries with the highest decrease among United Arab Emirates and Saudi Arabia with − 2614%, -1019% respectively. Across all countries, the most significant percentage changes in cancer deaths attributed to BMI were observed for pancreatic cancer, uterine cancer, and kidney cancer with uptrend increases in DALYs, deaths, ASDR, and ASMR from 1990 to 2019. Notably, pancreatic cancer in the United Arab Emirates, and Saudi Arabia saw the highest percentage increase in DALYs (2292%, 1654% respectively) and deaths (2052%, 1455% respectively) attributed to BMI during this period. Additionally, pancreatic cancer in Saudi Arabia experienced the highest percentage increase in ASDR (420.3%) and ASMR (442.1%). For uterine cancer, across all countries, the most significant percentage changes in uterine cancer deaths attributed to high BMI was in the United Arab Emirates, and Saudi Arabia with percentage increase in DALYs (794.5%, 528.7% respectively) and deaths (663.9%, 433.6% respectively) attributed to BMI during this period (Supplementary Table 1 A, Fig. 3).

### DALYs and Deaths of Specific Cancer Type Attributable to High BMI in 2019 and Percentage Change from 1990 to 2019 by Cancer Types and GCC Countries (Male)

In GBD 2019, 10 cancer types were found to be affected by high BMI among males, including esophageal cancer, gallbladder and biliary tract cancer, liver cancer, pancreatic cancer, multiple myeloma, colon and rectum cancer, thyroid cancer, kidney cancer, non-Hodgkin lymphoma, and leukemia. Across all countries, the most significant percentage changes in cancer deaths attributed to BMI in males were observed for colon and rectum cancer, esophageal cancer, kidney cancer and liver cancer with uptrend increases in DALYs, deaths, ASDR, and ASMR from 1990 to 2019. Notably, colon and rectum cancer deaths and DALY’s attributed to BMI in United Arab Emirates, and Qatar saw the highest percentage increase in DALYs (1423%, 1194% respectively) and deaths (1361%, 1226% respectively) attributed to BMI during this period. Oman experienced the highest percentage increase in colon and rectum cancer deaths attributed to BMI ASDR (222.5%) and ASMR (291.2%). Additionally, esophageal cancer deaths and DALY’s attributed to BMI in United Arab Emirates, and Qatar saw the highest percentage increase in DALYs (1811%, 733% respectively), and deaths (1758%, 750.7% respectively) during the study period (Supplementary Table 2B, Fig. 3).

### Association Between SDI and Burden of Cancer Attributable to High BMI

**Fig. 4:**
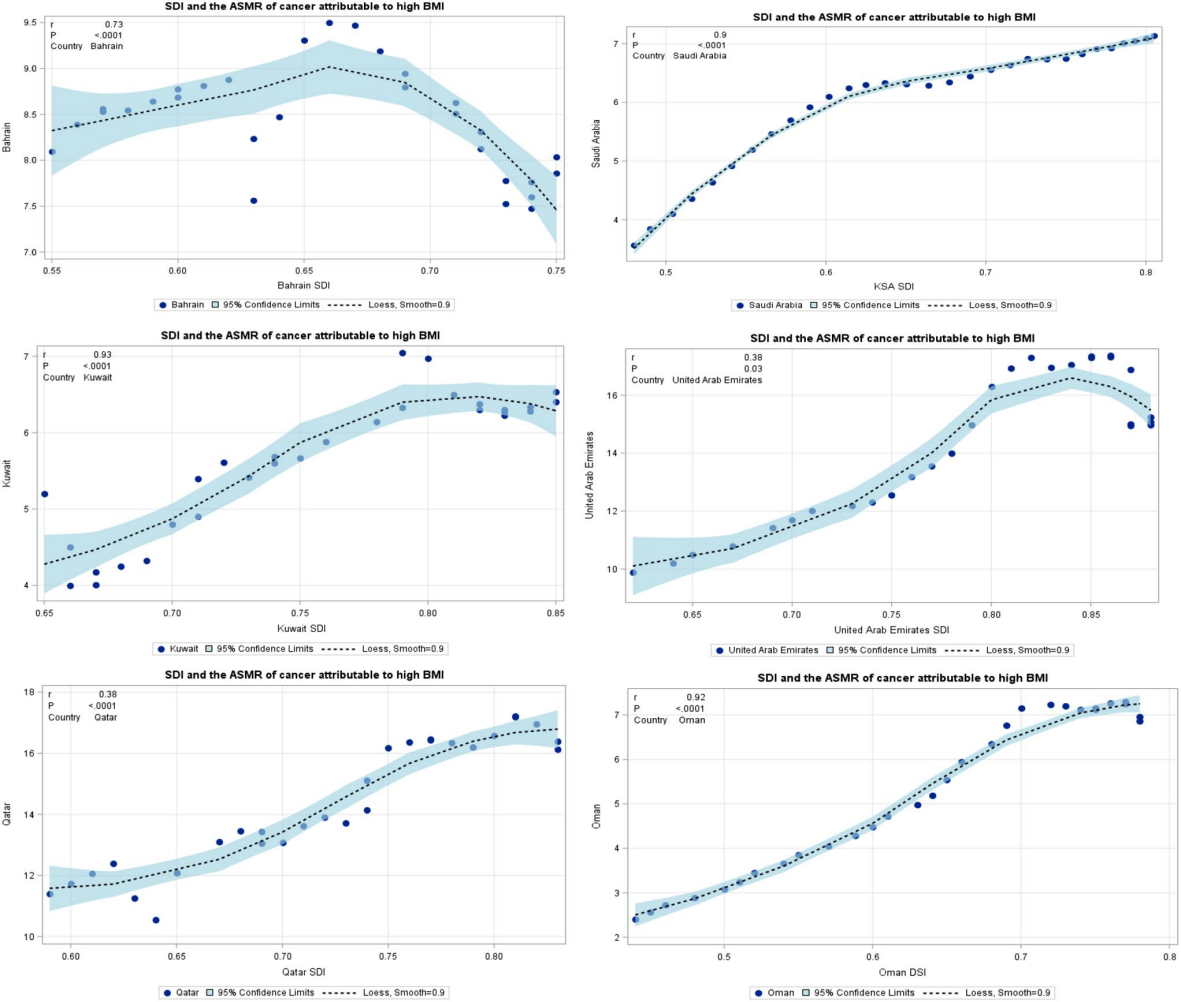
The association between SDI and the ASMR of cancer attributable to high BMI in 2019. Spearman’s correlation analysis reflected a significantly positive correlation between the ASMR and SDI level. Only Bahrain show significantly negative correlation was found between the ASMR and SDI level. ASMR, age-standardized mortality rate; BMI, body mass index; SDI, Socio-demographic Index

The SDI-based analysis showed that the death rate of cancer attributable to high BMI increased along with the increase in SDI level. In addition, The ASMR of cancer attributable to high BMI has increased in all GCC countries except for Bahrain which showed a decline in ASMR of cancer attributable to high BMI. The associations reflected a significantly positive correlation between the ASMR and SDI level in Saudi Arabia (*R* = 0.99, *p* < 0.0001), followed by Qatar and Oman with a similar correlation (*R* = 0.92, *p* < 0.0001). Only Bahrain showed a significantly negative weak correlation (*R*= -0.38, *p* = 0.03; Fig. 4).

## Discussion

Our findings reveal that in 2019, the GCC countries experienced over 102,206 cancer-related deaths. Among these, 2,210 (2.16%) deaths were attributed to high BMI, with an ASMR of 10 per 100,000. Notably, the ASMR associated with high BMI exceeded that of high socio-demographic countries (estimated at 7.52 per 100,000) and higher than the global ASMR (estimated at 5.69 per 100,000) for the same period [29–30]. Additionally, the collective burden of cancer-related DALYs amounted to 322,168 thousand. Of this total, 1.28% (4152 thousand) was attributed to high BMI, corresponding to an ASDR of 213.254 per 100,000 in 2019. Again, this ASDR surpassed that of high socio-demographic countries (ASDR: 174.68 per 100,000) and the global average (ASDR: 133.93 per 100,000). [29–30] This could be attributed to the ongoing exponential rise of obesity in GCC countries. Indeed, due to the significant modernization and adoption of the “Western lifestyle” in the last three decades, GCC countries are among the regions with the highest prevalence of obesity globally and even higher than high-income countries [4, 31–32]. These findings offer important information that can guide policymakers in creating effective prevention programs. Strategies aimed at reducing weight and encouraging physical activity could greatly lessen the impact of diseases in the GCC countries.

It is evident in previous studies that regions with higher SDI levels tended to carry a heavier burden of cancer attributed to high BMI [29–30]. Notably, the GCC countries, were all categorized as high SDI countries, except for Bahrain classified as a middle high SDI country. This trend was reflected in our findings, with Bahrain being the only country showing fluctuations over the years, including a slight decline in terms of death numbers, DALYs, ASDR, and ASMR between 1990 and 2019. In contrast, the United Arab Emirates experienced a substantial surge in DALYs attributed to cancer related to high BMI, increasing approximately 16-fold, particularly driven by a notable rise in DALYs among males, which highlight a concerning trend in mortality rates in the UAE. These findings also confirm the commonality of the population trend of high BMI condition on high income countries including GCC countries. This alarming increase requires tremendous all levels efforts to curb the associated outcomes of prevalent obesity. In fact, high BMI has not shown a declining trend but has instead continued to rise, contributing to its ongoing global pandemic. As of 2016, approximately 40% of adults and 18% of children worldwide were classified as obese or overweight, highlighting the persistent and widespread nature of this public health issue. This suggests that the continuation of current patterns of population weight gain will lead to continuing increases in the future burden of cancer in high income countries including the gulf region.

The GCC countries have experienced an increase in cancer cases over recent years, posing a significant public health challenge [9–10, 21]. Our findings highlight a distinctive pattern where specific cancers linked to high BMI, such as colon, rectal, and liver cancers, showed higher DALYs and mortality rates in the GCC compared to global trends. For instance, the DALYS and mortality rate for liver cancer in the GCC are notably higher, with ASMR and ASDR around six times and four times respectively higher than the global rate. Moreover, colon and esophageal cancer in the GCC are higher, with ASMR and ASDR around three times higher to the global rate [29]. All the above could explain the findings of increased deaths and the burden of cancer attributable to high BMI in GCC countries. On the other hand, our study reveals a consistent decrease in breast cancer DALYs and deaths among females, with the most substantial reductions observed in the United Arab Emirates and Saudi Arabia. This noteworthy decline may be attributed to heightened awareness campaigns and programs leading to improved early detection practices. Additionally, advancements in therapy and healthcare interventions in recent years likely play a crucial role in the observed decrease in the overall burden of breast cancer in the specified countries [33].

In 2019, across GCC countries, pancreatic cancer, uterine cancer, and kidney cancer accounted for 87.91% of the total attributable deaths associated with high BMI in female. In contrast in males, colon and rectum cancer alone accounted for 26% of all attributable deaths associated with high BMI. Combined colon and rectum cancer, esophageal cancer, kidney cancer and liver cancer accounted for 70% of all attributable deaths associated with high BMI. If we assume a causal relationship between high BMI and cancer, maintaining current trends of population weight gain will likely result in ongoing increases in the future burden of cancer. Significantly, approximately one-quarter of all cases attributed to high BMI (118,000 cancers) might have been preventable if the global population’s average BMI had stayed consistent with 1982 levels [23]. These findings provide policymakers with actionable data to inform evidence-based decision-making, prioritize resource allocation, and develop targeted interventions aimed at reducing the burden of cancer associated with high BMI. By addressing risk factors associated with specific cancer types, such as colon and rectum cancer in males and pancreatic, uterine, and kidney cancer in females, policymakers can tailor their efforts to reduce the burden of these diseases effectively.

Despite the higher prevalence and incidence of obesity among females compared to males in GCC countries [31, 34], persistent gender disparities are evident in the cancer burden associated with high BMI, with males having a greater disease burden and mortality related to high BMI. This observation warrants exploration, and one potential explanation is the differential impact of obesity on cancer types between genders. Specifically, certain cancer types associated with obesity, such as colon and rectum cancer, may be more prevalent and fatal in males than in females. This disparity in cancer-specific outcomes could contribute to the overall higher disease burden and mortality observed in males with obesity-related cancers. Another reason could be the difference in the biological nature of obesity in males and females. Males tend to have a higher prevalence of abdominal obesity, characterized by excess fat around the abdomen, which is particularly linked to an increased risk of more deadly diseases such as cardiovascular diseases [35–37] Abdominal obesity is associated with insulin resistance, dyslipidemia, hypertension, and inflammation, all of which contribute to the development and progression of cardiovascular diseases [38–39]. This might also contribute to the increased cancer burden associated with high BMI in males. Further research is needed to elucidate the underlying mechanisms driving these gender disparities and to develop targeted interventions aimed at reducing the cancer burden associated with obesity in both males and females.

### Limitations

This study has several potential limitations to consider. Firstly, population-level data may introduce ecological fallacy bias by incorrectly assuming that patterns or characteristics observed at the population level are true for individuals within that population. Secondly, while BMI is widely used, it doesn’t account for the percentage of body fat and different patterns of obesity [40–41]. This means that categorizing BMI may not accurately reflect the impact of high BMI on cancer in specific populations. Thirdly, the process of cancer development involves various factors, and this study doesn’t account for other potential influences that may interact with high BMI such as family history of cancer, introducing the possibility of confounding bias that could affect the results. Finally, in GBD 2019, data on high BMI prevalence and cancer mortality were collected for the same year, making it challenging to assess any time interval between exposure and the development of cancer and mortality.

## Conclusion

Our study highlights the significant impact of high BMI on cancer burden in GCC countries, revealing that in 2019, over 102,206 cancer-related fatalities occurred, with 2,210 (2.16%) attributed to high BMI, surpassing ASMRs of both high socio-demographic countries and the global average. This is concerning given the escalating rates of obesity in the region, driven by modernization and Western lifestyle adoption. Particularly alarming is the 16-fold increase in DALYs attributed to high BMI in the United Arab Emirates, emphasizing a pressing need for intervention. Moreover, the study identifies specific cancers, such as pancreatic, uterine, and kidney cancer in females, and colon and rectum cancer in males, as major contributors to attributable deaths, urging targeted prevention strategies. This study provides valuable insights into the contribution of high BMI to the burden of cancer in the GCC countries, one of the regions globally with the highest prevalence of obesity. These findings offer important information that can guide policymakers in creating effective prevention programs. Strategies aimed at reducing weight and encouraging physical activity could greatly lessen the impact of diseases in the GCC countries.

### Electronic Supplementary Material

Below is the link to the electronic supplementary material.

Supplementary Material 1

## Data Availability

The data that support the findings of this study are available from [Global Burden of Disease Study 2019]. No restrictions apply to the availability of these data, which were used under Public Use Files (PUF) data. Data are available at https://vizhub.healthdata.org.

## Abbreviations

ASDR: Age-standardized DALYs rate
ASMR: Age-standardized mortality rate
ASR: Age-standardized rate
BMI: Body mass index
DALYs: Disability-adjusted life-years
APC: Annual percentage change
PAFs: Population attribution fraction
GBD: Global Burden of Diseases
SDI: Socio-Demographic Index
UI: Uncertainty interval
ICD: International Classification of Diseases

## Declarations

All authors confirm that they had full access to all the data in the study and accept responsibility to submit for publication.
