# Supplementary material for: The Burden Cancer-Related Deaths Attributable to High Body Mass Index in a Gulf Cooperation Council: Results from the Global Burden of Disease Study 2019
Source: J Epidemiol Glob Health. 2024 May 13;14(2):379–97. doi: 10.1007/s44197-024-00241-5 (PMC11176139; doi:10.1007/s44197-024-00241-5)
Supplement: Supplementary file 1 — Supplementary Material 1 [file 44197_2024_241_MOESM1_ESM.docx]

**Supplementary Table 1A: Burden of DALYs and Deaths of cancer attributable to high BMI in 2019 and percentage change from 1990 to 2019 by cancer types and GCC countries (Females)**

| **Countries** | **Cancer types** | **Number of DALYs - Female** | | | **Number of Deaths - Female** | | | **ASDR per 100,00 - Female** | | | **ASMR per 100,00 - Female** | | |
| --- | --- | --- | --- | --- | --- | --- | --- | --- | --- | --- | --- | --- | --- |
|  |  | **1990**  **95% UI** | **2019**  **95% UI** | **% change** | **1990**  **95% UI** | **2019**  **95% UI** | **% change** | **1990**  **95% UI** | **2019**  **95% UI** | **% change** | **1990**  **95% UI** | **2019**  **95% UI** | **% change** |
| **Saudi Arabia** | *Breast cancer* | -315.76  (-864.18 , 46.32) | -3532.27  (-8304.16 , -290.43) | -1019 | -0.14  (-13.14 , 10.79) | -26.95  (-134.37 , 51.22) | -18897 | 1.41  (-12.99 , 13.92) | 7.56  (0.84 , 35.1) | 436.6 | 0.32  (-0.1 , 0.84) | 0.9  (-0.06 , 1.93) | 183.4 |
|  | *Colon and rectum cancer* | 266.46  (102.79 , 510.91) | 2308.31  (1038.65 , 3852.06) | 766.3 | 9.3  (3.54 , 18.3) | 69.2  (31.15 , 113.87) | 643.7 | 9.49  (3.64 , 18.35) | 23.06  (0.77 , 38) | 143 | 0.39  (0.15 , 0.77) | 0.94  (0.43 , 1.57) | 143 |
|  | *Esophageal cancer* | 341.48  (18.95 , 735.33) | 1209.31  (76.49 , 2322.75) | 254.1 | 12.73  (0.7 , 27.6) | 39.87  (2.45 , 75.6) | 213.2 | 13.06  (0.73 , 28.33) | 13.98  (1.19 , 26.47) | 7.05 | 0.55  (0.03 , 1.19) | 0.63  (0.04 , 1.21) | 13.76 |
|  | *Gallbladder and biliary tract cancer* | 332.66  (159.08 , 729.42) | 1568.59  (917.69 , 2497.08) | 371.5 | 12.99  (6.25 , 27.26) | 52.73  (31.38 , 84.74) | 306 | 13.04  (6.23 , 27.74) | 18.6  (1.19 , 30.18) | 42.59 | 0.58  (0.28 , 1.19) | 0.84  (0.49 , 1.34) | 44.48 |
|  | *Kidney cancer* | 115.72  (62.06 , 191.21) | 883.3  (523.92 , 1350.53) | 663.3 | 3.77  (1.95 , 6.4) | 23.24  (13.97 , 34.36) | 516.2 | 4.04  (2.12 , 6.79) | 8.23  (0.26 , 12.19) | 103.9 | 0.15  (0.08 , 0.26) | 0.29  (0.18 , 0.43) | 90.95 |
|  | *Leukemia* | 428.22  (163.22 , 881.15) | 1982.93  (837.2 , 3487.45) | 363.1 | 11.7  (4.44 , 23.59) | 50.09  (21.21 , 86.54) | 328.3 | 11.96  (4.56 , 24.25) | 15.89  (0.8 , 26.98) | 32.91 | 0.4  (0.15 , 0.8) | 0.55  (0.23 , 0.95) | 35.54 |
|  | *Liver cancer* | 249.8  (47 , 534.26) | 1076.1  (201.71 , 2103.2) | 330.8 | 9.08  (1.7 , 19.47) | 35.5  (6.64 , 69.29) | 291.2 | 9.23  (1.78 , 19.9) | 12.67  (0.84 , 25.09) | 37.27 | 0.39  (0.07 , 0.84) | 0.54  (0.1 , 1.09) | 40.89 |
|  | *Multiple myeloma* | 51.27  (15.55 , 106.64) | 274.08  (91.93 , 520.73) | 434.6 | 1.86  (0.57 , 4.01) | 8.78  (2.99 , 16.41) | 370.7 | 1.97  (0.6 , 4.17) | 3.15  (0.17 , 5.86) | 59.32 | 0.08  (0.02 , 0.17) | 0.13  (0.04 , 0.24) | 61.05 |
|  | *Non-Hodgkin lymphoma* | 279.35  (32.44 , 679.11) | 1580.08  (245.89 , 3206.92) | 465.6 | 8.86  (1.03 , 21.43) | 45.32  (7.28 , 91.46) | 411.6 | 8.89  (1.03 , 21.6) | 15.7  (0.84 , 31.83) | 76.64 | 0.35  (0.04 , 0.84) | 0.62  (0.09 , 1.27) | 79.58 |
|  | *Ovarian cancer* | 71.94  (-2.16 , 175.99) | 867.63  (-16.59 , 2111.96) | 1106 | 2.14  (-0.07 , 5.26) | 23.66  (-0.45 , 56.92) | 1003 | 2.35  (-0.07 , 5.73) | 7.56  (0.19 , 18.24) | 221.5 | 0.08  (0 , 0.19) | 0.26  (0 , 0.6) | 225.2 |
|  | *Pancreatic cancer* | 84.07  (27.06 , 170.59) | 1474.76  (549.74 , 2604.07) | 1654 | 3.09  (1.01 , 6.29) | 48.02  (17.88 , 84.77) | 1455 | 3.19  (1.04 , 6.52) | 16.61  (0.27 , 29.35) | 420.3 | 0.13  (0.04 , 0.27) | 0.72  (0.26 , 1.27) | 442.1 |
|  | *Thyroid cancer* | 74.35  (33.58 , 135.31) | 591.73  (327.31 , 1013.55) | 695.9 | 2.24  (1.01 , 4.35) | 12.55  (6.98 , 20.39) | 459.8 | 2.37  (1.07 , 4.6) | 5.11  (0.19 , 8.47) | 115.7 | 0.09  (0.04 , 0.19) | 0.16  (0.09 , 0.26) | 82.52 |
|  | *Uterine cancer* | 357.56  (195.89 , 592.78) | 2247.89  (1413.03 , 3197.2) | 528.7 | 12.53  (7.01 , 20.51) | 66.87  (42.12 , 92.21) | 433.6 | 13.36  (7.46 , 21.93) | 25.22  (0.87 , 35.8) | 88.74 | 0.53  (0.3 , 0.87) | 0.99  (0.58 , 1.4) | 85.91 |
|  | *Breast cancer* | -315.76  (-864.18 , 46.32) | -3532.27  (-8304.16 , -290.43) | 1019 | -0.14  (-13.14 , 10.79) | -26.95  (-134.37 , 51.22) | 18897 | 1.41  (-12.99 , 13.92) | 7.56  (0.84 , 35.1) | 436.6 | 0.32  (-0.1 , 0.84) | 0.9  (-0.06 , 1.93) | 183.4 |
|  | *Colon and rectum cancer* | 266.46  (102.79 , 510.91) | 2308.31  (1038.65 , 3852.06) | 766.3 | 9.3  (3.54 , 18.3) | 69.2  (31.15 , 113.87) | 643.7 | 9.49  (3.64 , 18.35) | 23.06  (0.77 , 38) | 143 | 0.39  (0.15 , 0.77) | 0.94  (0.43 , 1.57) | 143 |
| **Bahrain** | *Breast cancer* | -2.27  (-41.02 , 38.21) | -48.63  (-257.08 , 115.31) | -2043 | 0.68  (-0.45 , 2) | 2.82  (-2.93 , 8.66) | 203 | 23.52  (-14.19 , 67.12) | 20.97  (3.39 , 59.95) | -10.84 | 1.55  (0.15 , 3.39) | 1.8  (0.3 , 3.6) | 15.77 |
|  | *Colon and rectum cancer* | 16.06  (6.77 , 27.68) | 76.21  (33.76 , 128.62) | 374.5 | 0.53  (0.22 , 0.92) | 2.6  (1.17 , 4.39) | 511.6 | 16.9  (7.16 , 29.22) | 17.15  (1.23 , 29.02) | 1.47 | 0.7  (0.3 , 1.23) | 0.8  (0.36 , 1.35) | 14.36 |
|  | *Esophageal cancer* | 19.6  (0.9 , 39.67) | 30.84  (1.78 , 58.5) | 57.36 | 0.72  (0.03 , 1.46) | 1.15  (0.07 , 2.19) | 270.6 | 23.04  (1.07 , 46.56) | 7.57  (2 , 14.6) | -67.14 | 0.99  (0.05 , 2) | 0.36  (0.02 , 0.7) | -63.51 |
|  | *Gallbladder and biliary tract cancer* | 10  (5.46 , 15.64) | 36.83  (20.37 , 57.76) | 268.4 | 0.39  (0.21 , 0.61) | 1.44  (0.8 , 2.23) | 456.3 | 12.31  (6.68 , 19.22) | 9.63  (0.9 , 14.81) | -21.77 | 0.57  (0.3 , 0.9) | 0.48  (0.27 , 0.74) | -16.33 |
|  | *Kidney cancer* | 8.83  (5.37 , 13.2) | 51.08  (30.46 , 75.35) | 478.1 | 0.29  (0.18 , 0.43) | 1.6  (0.96 , 2.33) | 431.5 | 9.19  (5.57 , 13.61) | 10.86  (0.54 , 15.79) | 18.19 | 0.37  (0.23 , 0.54) | 0.46  (0.27 , 0.67) | 23.01 |
|  | *Leukemia* | 25.38  (10.19 , 45.05) | 70.82  (29.51 , 123.65) | 179 | 0.69  (0.27 , 1.21) | 2.09  (0.87 , 3.64) | 58.42 | 20.67  (8.19 , 36.38) | 14.83  (1.37 , 26.01) | -28.27 | 0.77  (0.31 , 1.37) | 0.6  (0.25 , 1.05) | -22.53 |
|  | *Liver cancer* | 13.85  (2.89 , 27.45) | 59.72  (10.67 , 120.82) | 331.2 | 0.51  (0.1 , 1.01) | 2.29  (0.42 , 4.63) | 344.5 | 16.09  (3.15 , 31.91) | 15.37  (1.48 , 31.21) | -4.48 | 0.74  (0.14 , 1.48) | 0.78  (0.14 , 1.55) | 4.85 |
|  | *Multiple myeloma* | 4.07  (1.31 , 7.83) | 17.46  (5.57 , 33.98) | 328.6 | 0.15  (0.05 , 0.28) | 0.63  (0.21 , 1.23) | 314 | 4.73  (1.5 , 9.04) | 4.1  (0.37 , 7.96) | -13.38 | 0.2  (0.06 , 0.37) | 0.18  (0.06 , 0.35) | -8.32 |
|  | *Non-Hodgkin lymphoma* | 6.69  (1.19 , 13.49) | 33.3  (5.54 , 68.99) | 397.9 | 0.21  (0.04 , 0.42) | 1.11  (0.18 , 2.33) | 389.9 | 6.38  (1.08 , 12.79) | 7.76  (0.52 , 16.26) | 21.64 | 0.26  (0.04 , 0.52) | 0.34  (0.06 , 0.72) | 32.41 |
|  | *Ovarian cancer* | 8.4  (-0.31 , 19.4) | 54.02  (-1.22 , 133.51) | 542.7 | 0.26  (-0.01 , 0.62) | 1.69  (-0.04 , 4.13) | 540.3 | 8.46  (-0.32 , 19.84) | 10.7  (0.76 , 25.85) | 26.4 | 0.32  (-0.01 , 0.76) | 0.43  (-0.01 , 1.02) | 32.7 |
|  | *Pancreatic cancer* | 11.43  (4.33 , 19.89) | 69.5  (26.7 , 121.88) | 507.9 | 0.42  (0.16 , 0.72) | 2.57  (0.96 , 4.57) | 373.3 | 13.34  (4.94 , 23.08) | 16.6  (1 , 29.51) | 24.46 | 0.58  (0.21 , 1) | 0.8  (0.3 , 1.42) | 36.96 |
|  | *Thyroid cancer* | 3.13  (1.61 , 5.09) | 15.81  (8.46 , 25.97) | 405.1 | 0.11  (0.06 , 0.19) | 0.53  (0.28 , 0.86) | 392.1 | 3.73  (1.94 , 6.14) | 4.15  (0.28 , 6.79) | 11.24 | 0.17  (0.09 , 0.28) | 0.19  (0.1 , 0.32) | 15.6 |
|  | *Uterine cancer* | 27.77  (17.79 , 40.56) | 145.53  (86.47 , 205.86) | 424 | 0.95  (0.6 , 1.4) | 4.67  (2.82 , 6.48) | 323.1 | 31.15  (19.8 , 46.04) | 31.96  (1.89 , 44.5) | 2.6 | 1.25  (0.79 , 1.89) | 1.32  (0.85 , 1.78) | 5.09 |
| **Oman** | *Breast cancer* | 2.3  (-32.01 , 36.72) | -2.62  (-220.55 , 215.18) | -213.9 | 0.65  (-0.27 , 1.94) | 5.05  (-1.68 , 12.97) | 672.4 | 5.54  (-3.34 , 17.37) | 23.58  (0.8 , 54.36) | 326 | 0.33  (0.01 , 0.8) | 1.3  (0.35 , 2.57) | 298.9 |
|  | *Colon and rectum cancer* | 22.78  (8.58 , 47.25) | 135.7  (61.97 , 242.99) | 495.8 | 0.8  (0.29 , 1.69) | 5.04  (2.3 , 8.89) | 526.7 | 6.95  (2.56 , 14.53) | 17.48  (0.59 , 30.88) | 151.4 | 0.28  (0.1 , 0.59) | 0.82  (0.37 , 1.44) | 194.5 |
|  | *Esophageal cancer* | 31.14  (1.54 , 71.6) | 104.57  (5.72 , 201.26) | 235.8 | 1.13  (0.06 , 2.68) | 4.01  (0.22 , 7.82) | 253.4 | 9.77  (0.49 , 22.64) | 13.84  (0.95 , 27.31) | 41.65 | 0.39  (0.02 , 0.95) | 0.64  (0.04 , 1.28) | 64.46 |
|  | *Gallbladder and biliary tract cancer* | 22.99  (10.8 , 40.73) | 71.26  (40.17 , 112.25) | 209.9 | 0.88  (0.4 , 1.56) | 2.84  (1.59 , 4.47) | 222.6 | 7.42  (3.43 , 13.2) | 9.81  (0.56 , 15.34) | 32.22 | 0.31  (0.14 , 0.56) | 0.48  (0.26 , 0.75) | 52.01 |
|  | *Kidney cancer* | 9.62  (4.47 , 16.9) | 71.34  (43.32 , 103.86) | 641.8 | 0.32  (0.15 , 0.56) | 2.23  (1.37 , 3.22) | 603.4 | 2.83  (1.33 , 5.03) | 7.97  (0.19 , 11.54) | 181.6 | 0.1  (0.05 , 0.19) | 0.31  (0.19 , 0.46) | 200.5 |
|  | *Leukemia* | 36.01  (13.18 , 72.84) | 149.06  (59.35 , 266.55) | 313.9 | 1.05  (0.38 , 2.13) | 4.36  (1.75 , 7.85) | 314 | 9.28  (3.38 , 18.58) | 14.61  (0.65 , 26.29) | 57.42 | 0.32  (0.11 , 0.65) | 0.58  (0.23 , 1.02) | 80.37 |
|  | *Liver cancer* | 20.01  (3.2 , 45.93) | 95.3  (18.14 , 189.11) | 376.2 | 0.71  (0.11 , 1.61) | 3.38  (0.64 , 6.51) | 378.6 | 6.08  (0.96 , 14.06) | 11.52  (0.55 , 22.25) | 89.58 | 0.24  (0.04 , 0.55) | 0.5  (0.09 , 0.96) | 110.1 |
|  | *Multiple myeloma* | 5.91  (1.66 , 12.4) | 30.16  (10.33 , 57.48) | 410.2 | 0.21  (0.06 , 0.44) | 1.1  (0.37 , 2.11) | 417.1 | 1.85  (0.52 , 3.89) | 3.83  (0.15 , 7.33) | 107.1 | 0.07  (0.02 , 0.15) | 0.16  (0.06 , 0.32) | 127.4 |
|  | *Non-Hodgkin lymphoma* | 18.69  (2.57 , 46.32) | 124.82  (20.15 , 260.82) | 567.8 | 0.61  (0.09 , 1.52) | 4.06  (0.65 , 8.29) | 570.1 | 5.28  (0.74 , 13.07) | 13.9  (0.5 , 28.64) | 163.4 | 0.2  (0.03 , 0.5) | 0.6  (0.1 , 1.24) | 202 |
|  | *Ovarian cancer* | 5.23  (-0.12 , 13.84) | 65.38  (-1.39 , 155.14) | 1150 | 0.16  (0 , 0.43) | 2.03  (-0.05 , 4.84) | 1148 | 1.48  (-0.03 , 3.94) | 6.94  (0.14 , 16.64) | 368.5 | 0.05  (0 , 0.14) | 0.25  (-0.01 , 0.6) | 404.7 |
|  | *Pancreatic cancer* | 8.48  (2.66 , 17.93) | 125.1  (48.78 , 214.94) | 1376 | 0.31  (0.09 , 0.66) | 4.8  (1.85 , 8.21) | 1445 | 2.66  (0.81 , 5.65) | 16.4  (0.23 , 28.03) | 516.8 | 0.11  (0.03 , 0.23) | 0.75  (0.28 , 1.31) | 603.5 |
|  | *Thyroid cancer* | 4.89  (2.24 , 8.92) | 27.04  (14.43 , 44.04) | 453.3 | 0.15  (0.07 , 0.27) | 0.71  (0.39 , 1.12) | 385 | 1.37  (0.63 , 2.49) | 2.87  (0.09 , 4.59) | 110.4 | 0.05  (0.02 , 0.09) | 0.1  (0.06 , 0.17) | 117.7 |
|  | *Uterine cancer* | 23.75  (12.94 , 38.84) | 100.77  (65.08 , 139.42) | 324.3 | 0.83  (0.44 , 1.36) | 3.45  (2.2 , 4.75) | 313.6 | 7.42  (3.95 , 12.1) | 12.84  (0.47 , 17.73) | 72.99 | 0.29  (0.14 , 0.47) | 0.53  (0.33 , 0.73) | 86.07 |
| **Kuwait** | *Breast cancer* | -62.74  (-154.45 , 9.79) | -165.75  (-588.33 , 153.26) | -164.2 | 0.26  (-2.1 , 2.46) | 3.79  (-7.14 , 13.91) | 1377 | 16.06  (-10.61 , 44.32) | 20.54  (2.2 , 46.12) | 27.9 | 1.09  (0.09 , 2.2) | 1.24  (0.32 , 2.37) | 13.77 |
|  | *Colon and rectum cancer* | 41.87  (19.34 , 70.45) | 196.72  (91.26 , 334.93) | 369.8 | 1.45  (0.67 , 2.47) | 7.19  (3.33 , 12.3) | 396.7 | 15.34  (7.12 , 26.07) | 15.81  (1.13 , 26.87) | 3.05 | 0.65  (0.3 , 1.13) | 0.75  (0.34 , 1.29) | 15.62 |
|  | *Esophageal cancer* | 34.14  (1.92 , 66.31) | 79.99  (4.68 , 148.98) | 134.4 | 1.26  (0.08 , 2.49) | 3.12  (0.18 , 5.77) | 147.3 | 13.62  (0.81 , 27.03) | 6.95  (1.19 , 12.65) | -48.96 | 0.6  (0.04 , 1.19) | 0.35  (0.02 , 0.64) | -41.86 |
|  | *Gallbladder and biliary tract cancer* | 51.39  (29.39 , 76.6) | 113.63  (66.4 , 178.91) | 121.1 | 1.94  (1.09 , 2.9) | 4.42  (2.57 , 6.84) | 128.3 | 21.27  (12.03 , 31.79) | 10.03  (1.41 , 15.59) | -52.84 | 0.93  (0.53 , 1.41) | 0.49  (0.28 , 0.76) | -47.84 |
|  | *Kidney cancer* | 25.49  (16.3 , 37.1) | 112.49  (69.61 , 164.22) | 341.3 | 0.79  (0.5 , 1.16) | 3.5  (2.2 , 5.06) | 343.3 | 8.63  (5.37 , 12.75) | 7.95  (0.51 , 11.57) | -7.87 | 0.33  (0.2 , 0.51) | 0.33  (0.21 , 0.47) | -1.08 |
|  | *Leukemia* | 78.23  (32.48 , 133.82) | 177.37  (80.14 , 306.64) | 126.7 | 2.09  (0.86 , 3.56) | 5.19  (2.4 , 9.02) | 148 | 20.26  (8.21 , 34.97) | 11.07  (1.26 , 18.98) | -45.35 | 0.73  (0.3 , 1.26) | 0.44  (0.2 , 0.75) | -40.05 |
|  | *Liver cancer* | 20.56  (3.97 , 39.92) | 72.42  (15.47 , 140.73) | 252.3 | 0.7  (0.13 , 1.39) | 2.63  (0.56 , 5.08) | 275 | 7.56  (1.41 , 14.99) | 6.05  (0.64 , 11.6) | -19.9 | 0.31  (0.05 , 0.64) | 0.28  (0.06 , 0.54) | -10.79 |
|  | *Multiple myeloma* | 8.05  (2.8 , 14.81) | 40.35  (13.21 , 78.08) | 401.6 | 0.28  (0.09 , 0.52) | 1.32  (0.43 , 2.51) | 377.1 | 3.15  (1.06 , 5.85) | 3.11  (0.24 , 5.91) | -1.2 | 0.13  (0.04 , 0.24) | 0.13  (0.04 , 0.24) | -0.07 |
|  | *Non-Hodgkin lymphoma* | 25.43  (4.49 , 52.88) | 82.52  (13.31 , 165) | 224.5 | 0.77  (0.14 , 1.61) | 2.64  (0.43 , 5.26) | 241.3 | 8.16  (1.45 , 17.06) | 6  (0.66 , 11.87) | -26.39 | 0.31  (0.06 , 0.66) | 0.25  (0.04 , 0.49) | -20.14 |
|  | *Ovarian cancer* | 21.09  (-0.51 , 48.49) | 95.76  (-2.32 , 224.3) | 354 | 0.62  (-0.02 , 1.42) | 2.9  (-0.07 , 6.76) | 367.8 | 6.7  (-0.16 , 15.24) | 6.45  (0.56 , 14.9) | -3.73 | 0.24  (-0.01 , 0.56) | 0.25  (-0.01 , 0.57) | 2.74 |
|  | *Pancreatic cancer* | 26.86  (10.28 , 46.48) | 147.99  (57.68 , 245.25) | 451.1 | 1.01  (0.38 , 1.77) | 5.79  (2.27 , 9.68) | 476.1 | 10.93  (4.13 , 19.16) | 13  (0.84 , 21.74) | 18.96 | 0.48  (0.18 , 0.84) | 0.63  (0.25 , 1.06) | 32.25 |
|  | *Thyroid cancer* | 12.99  (7.39 , 19.83) | 37.96  (21.52 , 59.69) | 192.3 | 0.38  (0.22 , 0.59) | 1.12  (0.63 , 1.74) | 192.2 | 4.6  (2.6 , 7) | 3.13  (0.28 , 4.85) | -32.05 | 0.18  (0.1 , 0.28) | 0.13  (0.07 , 0.2) | -26.9 |
|  | *Uterine cancer* | 68.84  (49.14 , 90.2) | 373.8  (262.67 , 504.44) | 443 | 2.36  (1.67 , 3.12) | 12.86  (8.92 , 17.36) | 445.3 | 27.21  (19.4 , 35.99) | 31.65  (1.47 , 42.92) | 16.33 | 1.11  (0.78 , 1.47) | 1.38  (0.96 , 1.89) | 25.16 |
| **Qatar** | *Breast cancer* | -24.52  (-56.22 , 0.16) | -161.22  (-413.23 , 22.67) | -557.4 | -0.13  (-0.91 , 0.55) | 0.24  (-5.53 , 5.2) | -289.7 | 30.2  (-14.32 , 80.54) | 65.94  (4.09 , 136.58) | 118.4 | 1.93  (0.24 , 4.09) | 4.21  (1.37 , 7.6) | 118.5 |
|  | *Colon and rectum cancer* | 14.02  (6.16 , 23.81) | 106.99  (48.25 , 183.53) | 663 | 0.47  (0.21 , 0.83) | 3.51  (1.62 , 5.95) | 649.9 | 31.62  (13.94 , 55.75) | 46.43  (2.52 , 77.04) | 46.81 | 1.42  (0.62 , 2.52) | 2.59  (1.22 , 4.25) | 82.54 |
|  | *Esophageal cancer* | 11.4  (0.7 , 22.13) | 45.25  (2.77 , 86.01) | 297.1 | 0.41  (0.02 , 0.79) | 1.64  (0.1 , 3.13) | 302 | 29.09  (1.53 , 55.9) | 23.18  (2.59 , 44.84) | -20.31 | 1.33  (0.07 , 2.59) | 1.33  (0.08 , 2.56) | 0.28 |
|  | *Gallbladder and biliary tract cancer* | 10.66  (5.65 , 16.13) | 41.37  (24.35 , 64.5) | 288.2 | 0.4  (0.21 , 0.62) | 1.57  (0.91 , 2.45) | 289.8 | 28.97  (14.83 , 44.98) | 24.08  (2.24 , 37.13) | -16.88 | 1.4  (0.69 , 2.24) | 1.45  (0.81 , 2.22) | 3.55 |
|  | *Kidney cancer* | 4.85  (2.9 , 7.38) | 37.97  (23.1 , 56.33) | 682.8 | 0.15  (0.09 , 0.22) | 1.11  (0.68 , 1.66) | 649.5 | 10.12  (5.8 , 15.35) | 13.62  (0.63 , 19.94) | 34.57 | 0.42  (0.23 , 0.63) | 0.66  (0.41 , 0.96) | 57.58 |
|  | *Leukemia* | 15.56  (6.18 , 28.67) | 81.64  (35.08 , 147.14) | 424.7 | 0.41  (0.17 , 0.73) | 2.18  (0.94 , 3.82) | 434.8 | 23.6  (10.2 , 41.9) | 25.29  (1.64 , 43.21) | 7.15 | 0.9  (0.37 , 1.64) | 1.22  (0.52 , 2.09) | 35.23 |
|  | *Liver cancer* | 17.88  (3.37 , 35.15) | 102.76  (19.09 , 196.65) | 474.8 | 0.69  (0.13 , 1.36) | 3.79  (0.7 , 7.25) | 451.8 | 51.25  (9.94 , 102.43) | 52.83  (4.82 , 98.61) | 3.08 | 2.4  (0.45 , 4.82) | 2.99  (0.58 , 5.56) | 24.51 |
|  | *Multiple myeloma* | 1.34  (0.44 , 2.48) | 9.6  (3.28 , 18.04) | 619.3 | 0.05  (0.02 , 0.08) | 0.32  (0.11 , 0.59) | 608 | 3.26  (1.12 , 6.17) | 3.91  (0.26 , 7.24) | 19.87 | 0.14  (0.05 , 0.26) | 0.19  (0.07 , 0.36) | 39.05 |
|  | *Non-Hodgkin lymphoma* | 6.48  (0.95 , 14.29) | 47.18  (7.39 , 97.83) | 627.7 | 0.18  (0.03 , 0.38) | 1.36  (0.21 , 2.86) | 650.9 | 11.13  (1.73 , 23.01) | 15.93  (0.92 , 32.78) | 43.16 | 0.43  (0.07 , 0.92) | 0.77  (0.13 , 1.56) | 78.11 |
|  | *Ovarian cancer* | 3.64  (-0.14 , 8.81) | 47.79  (-0.97 , 108) | 1212 | 0.11  (0 , 0.26) | 1.42  (-0.03 , 3.23) | 1188 | 7.45  (-0.26 , 18.22) | 14.03  (0.68 , 32.52) | 88.32 | 0.28  (-0.01 , 0.68) | 0.58  (-0.01 , 1.33) | 105.6 |
|  | *Pancreatic cancer* | 4.57  (1.59 , 8.08) | 64.7  (25.23 , 114.13) | 1315 | 0.16  (0.06 , 0.29) | 2.22  (0.87 , 3.86) | 1296 | 11.07  (3.86 , 20.6) | 27.8  (0.98 , 48.59) | 151.2 | 0.5  (0.17 , 0.98) | 1.49  (0.57 , 2.58) | 199.2 |
|  | *Thyroid cancer* | 1.73  (0.93 , 2.87) | 11.23  (6.06 , 19.8) | 549.6 | 0.05  (0.03 , 0.08) | 0.27  (0.15 , 0.44) | 430.2 | 3.56  (1.88 , 5.91) | 4.43  (0.26 , 7.14) | 24.2 | 0.15  (0.08 , 0.26) | 0.23  (0.13 , 0.37) | 49.52 |
|  | *Uterine cancer* | 9.49  (5.42 , 13.85) | 69.19  (31.27 , 101.38) | 629.2 | 0.33  (0.17 , 0.48) | 2.28  (0.93 , 3.34) | 598.8 | 24.53  (12.15 , 36.81) | 32.88  (1.6 , 47.97) | 34.03 | 1.03  (0.48 , 1.6) | 1.6  (0.6 , 2.33) | 55.39 |
| **United Arab Emirates** | *Breast cancer* | -44.5  (-136 , 33.62) | -1207.79  (-2805.33 , -55.7) | -2614 | 0.18  (-2.06 , 2.59) | -13.97  (-49.73 , 14.24) | -7782 | 30.1  (-6.99 , 79.1) | 32.61  (3.57 , 92.33) | 8.35 | 1.57  (0.2 , 3.57) | 2.04  (0.13 , 4.24) | 29.68 |
|  | *Colon and rectum cancer* | 52.65  (22.25 , 96.73) | 498.17  (220.65 , 889.44) | 846.2 | 1.62  (0.67 , 2.99) | 13.96  (6.04 , 25.35) | 763 | 28.71  (11.51 , 53.79) | 33.16  (2.26 , 64.95) | 15.49 | 1.18  (0.48 , 2.26) | 1.41  (0.55 , 2.9) | 20.12 |
|  | *Esophageal cancer* | 73.85  (2.82 , 174.08) | 545.51  (27.31 , 1243.45) | 638.7 | 2.38  (0.09 , 5.69) | 15.5  (0.76 , 35.24) | 551.1 | 43.02  (1.66 , 103.71) | 37.3  (4.52 , 86.59) | -13.3 | 1.83  (0.07 , 4.52) | 1.61  (0.07 , 3.82) | -12.06 |
|  | *Gallbladder and biliary tract cancer* | 43.13  (16.28 , 94.04) | 251.05  (110.12 , 485.06) | 482 | 1.46  (0.53 , 3.16) | 7.32  (3.19 , 14.3) | 403.4 | 26.85  (9.23 , 59.22) | 18.52  (2.62 , 36.16) | -31.01 | 1.19  (0.39 , 2.62) | 0.82  (0.32 , 1.64) | -30.68 |
|  | *Kidney cancer* | 21.55  (9.18 , 61.84) | 275.58  (118.04 , 700.16) | 1179 | 0.59  (0.24 , 1.73) | 6.66  (2.83 , 17.44) | 1036 | 9.78  (3.82 , 29.29) | 14.27  (1.16 , 39.93) | 45.94 | 0.37  (0.14 , 1.16) | 0.52  (0.21 , 1.54) | 40.81 |
|  | *Leukemia* | 87.65  (34.08 , 167.93) | 642.66  (276.38 , 1168.02) | 633.2 | 2.36  (0.91 , 4.57) | 16.5  (7 , 29.59) | 600.1 | 38.71  (14.85 , 74.04) | 33.57  (2.75 , 60.67) | -13.27 | 1.42  (0.53 , 2.75) | 1.22  (0.5 , 2.24) | -14.7 |
|  | *Liver cancer* | 22.2  (3.65 , 50.81) | 174.73  (30.95 , 372.93) | 687 | 0.68  (0.11 , 1.58) | 4.74  (0.85 , 10.21) | 599.4 | 11.67  (1.9 , 27.67) | 10.61  (1.2 , 24.05) | -9.12 | 0.49  (0.08 , 1.2) | 0.44  (0.07 , 1.05) | -10.53 |
|  | *Multiple myeloma* | 7.39  (1.74 , 18.66) | 64.63  (17.03 , 176.62) | 774.2 | 0.24  (0.05 , 0.61) | 1.81  (0.46 , 5.01) | 665.5 | 4.25  (0.95 , 10.99) | 4.22  (0.43 , 11.98) | -0.57 | 0.17  (0.04 , 0.43) | 0.17  (0.04 , 0.48) | -1.79 |
|  | *Non-Hodgkin lymphoma* | 30.03  (2.66 , 90.81) | 227.53  (20.58 , 554.36) | 657.8 | 0.78  (0.07 , 2.34) | 5.55  (0.47 , 13.78) | 613.6 | 11.63  (1 , 34.91) | 10.99  (1.27 , 28.03) | -5.43 | 0.42  (0.03 , 1.27) | 0.38  (0.03 , 1.01) | -9.33 |
|  | *Ovarian cancer* | 13.2  (-0.39 , 32.78) | 221.34  (-4.26 , 580.59) | 1576 | 0.35  (-0.01 , 0.85) | 5.61  (-0.11 , 14.84) | 1522 | 5.4  (-0.18 , 13.94) | 10.91  (0.48 , 28.62) | 101.9 | 0.18  (-0.01 , 0.48) | 0.38  (-0.01 , 1) | 107.5 |
|  | *Pancreatic cancer* | 25.86  (7.62 , 52.68) | 618.72  (175.88 , 1316.49) | 2292 | 0.84  (0.24 , 1.75) | 18.05  (5.09 , 38.57) | 2052 | 15.05  (4.26 , 31.55) | 44.13  (1.3 , 96.33) | 193.1 | 0.62  (0.17 , 1.3) | 1.89  (0.52 , 4.1) | 201.9 |
|  | *Thyroid cancer* | 7.78  (3.43 , 14.42) | 72.17  (30.96 , 137.08) | 827.2 | 0.2  (0.08 , 0.39) | 1.57  (0.66 , 3) | 670.3 | 3.58  (1.33 , 6.96) | 3.91  (0.27 , 7.57) | 9.22 | 0.14  (0.05 , 0.27) | 0.14  (0.05 , 0.27) | 1.16 |
|  | *Uterine cancer* | 45.42  (18.44 , 87.2) | 406.26  (183.93 , 674.24) | 794.5 | 1.39  (0.53 , 2.76) | 10.63  (4.54 , 18.28) | 663.9 | 25.4  (9.16 , 51.42) | 25.09  (2.11 , 45.27) | -1.25 | 1.01  (0.34 , 2.11) | 0.95  (0.36 , 1.8) | -5.23 |

*BMI, body mass index; GCC, gulf cooperation council; ASDR, age-standardized DALYs rate; ASMR, age-standardized mortality rate; DALYs, disability-adjusted life-years; UI, uncertainty interval*

**Supplementary Table 2B: Burden of DALYs and Deaths of cancer attributable to high BMI in 2019 and percentage change from 1990 to 2019 by cancer types and GCC countries (Males)**

| **Countries** | **cancer types** | **Number of DALYs - male** | | | **Number of Deaths - male** | | | **ASDR per 100,00 - male** | | | **ASMR per 100,00 - male** | | |
| --- | --- | --- | --- | --- | --- | --- | --- | --- | --- | --- | --- | --- | --- |
|  |  | **1990**  **95% UI** | **2019**  **95% UI** | **% change** | **1990**  **95% UI** | **2019**  **95% UI** | **% change** | **1990**  **95% UI** | **2019**  **95% UI** | **% change** | **1990**  **95% UI** | **2019**  **95% UI** | **% change** |
| **Saudi Arabia** | *Colon and rectum cancer* | 687.36  (310.59 , 1280.45) | 7136.55  (4106.93 , 10688.26) | 938.3 | 22.74  (10.28 , 41.84) | 212.22  (122.1 , 317.3) | 833.1 | 16.37  (7.38 , 30.14) | 47.95  (1.26 , 72.6) | 192.9 | 0.68  (0.3 , 1.26) | 1.98  (1.14 , 3.01) | 192.4 |
|  | *Esophageal cancer* | 383.08  (70.59 , 796.34) | 2437.04  (613.23 , 4508.34) | 536.2 | 13.32  (2.34 , 27.49) | 77.3  (19.74 , 141.38) | 480.3 | 9.71  (1.7 , 20) | 17.39  (0.83 , 31.39) | 78.98 | 0.4  (0.07 , 0.83) | 0.74  (0.19 , 1.35) | 83.85 |
|  | *Gallbladder and biliary tract cancer* | 110.26  (20.63 , 244.47) | 681.23  (126.87 , 1378.47) | 517.9 | 4.01  (0.76 , 8.88) | 22.56  (4.3 , 44.37) | 463.1 | 2.9  (0.55 , 6.44) | 5.24  (0.29 , 10.26) | 80.64 | 0.13  (0.02 , 0.29) | 0.24  (0.05 , 0.46) | 84.02 |
|  | *Kidney cancer* | 120.95  (49.75 , 233.35) | 2231.23  (1192.58 , 3614.22) | 1745 | 3.91  (1.58 , 7.66) | 66.45  (35.84 , 106.37) | 1600 | 2.82  (1.16 , 5.52) | 14.89  (0.22 , 23.47) | 428 | 0.11  (0.04 , 0.22) | 0.6  (0.33 , 0.94) | 431.5 |
|  | *Leukemia* | 328.11  (136.47 , 603.65) | 2159.79  (1052.05 , 3763) | 558.2 | 9.1  (3.76 , 17.09) | 53.79  (26.18 , 93.5) | 491.3 | 6.27  (2.61 , 11.82) | 11.05  (0.44 , 19.21) | 76.09 | 0.23  (0.09 , 0.44) | 0.39  (0.18 , 0.67) | 68.84 |
|  | *Liver cancer* | 1222.85  (378.95 , 2509.43) | 4677.55  (1819.7 , 8580.92) | 282.5 | 41.92  (13.14 , 84.5) | 161  (62.65 , 290.15) | 284.1 | 30.47  (9.57 , 61.48) | 38.36  (2.5 , 69.21) | 25.89 | 1.23  (0.38 , 2.5) | 1.69  (0.63 , 3.03) | 37.57 |
|  | *Multiple myeloma* | 53.58  (14.47 , 125.3) | 447.34  (127.45 , 932.73) | 734.9 | 1.82  (0.48 , 4.47) | 13.7  (3.93 , 27.96) | 652.4 | 1.33  (0.35 , 3.29) | 3.1  (0.13 , 6.33) | 132.3 | 0.05  (0.01 , 0.13) | 0.13  (0.04 , 0.26) | 131.7 |
|  | *Non-Hodgkin lymphoma* | 257.5  (85.11 , 551.36) | 1931.83  (769.59 , 3640.76) | 650.2 | 8.22  (2.73 , 17.75) | 52.88  (21.35 , 99.43) | 543.2 | 5.8  (1.92 , 12.55) | 11.67  (0.51 , 21.79) | 101.4 | 0.23  (0.08 , 0.51) | 0.45  (0.17 , 0.84) | 90.19 |
|  | *Pancreatic cancer* | 87.97  (-0.61 , 229.57) | 1666.73  (-12.14 , 3802.99) | 1795 | 3.08  (-0.02 , 8.05) | 51.98  (-0.38 , 116.09) | 1586 | 2.24  (-0.02 , 5.86) | 11.47  (0.25 , 25.74) | 412.4 | 0.09  (0 , 0.25) | 0.48  (0 , 1.07) | 410.6 |
|  | *Thyroid cancer* | 44.11  (12.98 , 94.5) | 652.48  (213.01 , 1178.62) | 1379 | 1.36  (0.39 , 2.97) | 16.12  (5.33 , 28.89) | 1088 | 0.99  (0.28 , 2.17) | 3.86  (0.09 , 6.9) | 290.1 | 0.04  (0.01 , 0.09) | 0.14  (0.04 , 0.24) | 250.8 |
| **Bahrain** | *Colon and rectum cancer* | 52.99  (28.12 , 84.16) | 338.95  (186.82 , 535.15) | 539.7 | 1.76  (0.93 , 2.81) | 11.09  (6.21 , 17.51) | 528.8 | 45.19  (23.52 , 71.73) | 47.69  (3.28 , 74.36) | 5.53 | 2.07  (1.04 , 3.28) | 2.38  (1.29 , 3.74) | 15.09 |
|  | *Esophageal cancer* | 34.47  (7.88 , 67.7) | 133.45  (32.31 , 246.38) | 287.1 | 1.27  (0.29 , 2.52) | 4.67  (1.14 , 8.65) | 269.2 | 33.55  (7.72 , 67.21) | 19.61  (3.21 , 36.17) | -41.55 | 1.58  (0.37 , 3.21) | 1.01  (0.23 , 1.91) | -36.07 |
|  | *Gallbladder and biliary tract cancer* | 5.01  (0.99 , 9.95) | 22.84  (4.57 , 44.88) | 356 | 0.18  (0.03 , 0.36) | 0.79  (0.16 , 1.56) | 341.3 | 4.72  (0.91 , 9.49) | 3.34  (0.46 , 6.54) | -29.36 | 0.22  (0.04 , 0.46) | 0.17  (0.04 , 0.35) | -22.29 |
|  | *Kidney cancer* | 17.03  (8.5 , 28.73) | 117.55  (63.45 , 183.49) | 590.1 | 0.58  (0.28 , 0.97) | 3.81  (2.04 , 5.99) | 559.9 | 14.74  (7.1 , 24.82) | 15.2  (1.07 , 24.32) | 3.12 | 0.64  (0.31 , 1.07) | 0.7  (0.37 , 1.14) | 10.3 |
|  | *Leukemia* | 24.72  (11.14 , 43) | 106.28  (50.08 , 186.94) | 329.9 | 0.67  (0.3 , 1.18) | 3.08  (1.43 , 5.3) | 356.2 | 15.18  (6.63 , 26.88) | 13.48  (1.17 , 22.73) | -11.17 | 0.65  (0.28 , 1.17) | 0.61  (0.27 , 1.04) | -5.95 |
|  | *Liver cancer* | 35.55  (11.94 , 66.85) | 214.3  (80.21 , 399.42) | 502.8 | 1.23  (0.42 , 2.33) | 7.25  (2.72 , 13.6) | 488.1 | 31.44  (10.89 , 59.75) | 29.12  (2.77 , 54.53) | -7.37 | 1.44  (0.48 , 2.77) | 1.42  (0.51 , 2.65) | -1.18 |
|  | *Multiple myeloma* | 5.26  (1.44 , 11.18) | 29.11  (7.94 , 67.06) | 452.9 | 0.19  (0.05 , 0.4) | 0.97  (0.27 , 2.18) | 419.9 | 4.91  (1.33 , 10.68) | 3.97  (0.5 , 8.78) | -19.07 | 0.23  (0.06 , 0.5) | 0.19  (0.05 , 0.4) | -15.42 |
|  | *Non-Hodgkin lymphoma* | 12.81  (4.53 , 24.6) | 81.05  (28.75 , 149.52) | 532.8 | 0.36  (0.13 , 0.68) | 2.31  (0.84 , 4.24) | 536.5 | 8.21  (2.88 , 15.43) | 9.79  (0.66 , 18.17) | 19.16 | 0.35  (0.12 , 0.66) | 0.42  (0.15 , 0.8) | 21.68 |
|  | *Pancreatic cancer* | 11.38  (-0.09 , 27.67) | 99.37  (-0.8 , 234.28) | 773.1 | 0.4  (0 , 0.97) | 3.41  (-0.03 , 8.09) | 749.8 | 10.29  (-0.08 , 24.78) | 13.95  (1.15 , 33) | 35.57 | 0.48  (0 , 1.15) | 0.7  (-0.01 , 1.62) | 46.06 |
|  | *Thyroid cancer* | 2.12  (0.63 , 4.17) | 13.89  (4.35 , 27.14) | 554.3 | 0.07  (0.02 , 0.14) | 0.41  (0.13 , 0.81) | 495.3 | 1.83  (0.56 , 3.63) | 1.88  (0.17 , 3.59) | 2.72 | 0.08  (0.03 , 0.17) | 0.09  (0.03 , 0.17) | 5.36 |
| **Oman** | *Colon and rectum cancer* | 55.02  (22.02 , 107.92) | 445.74  (242.05 , 769.98) | 710.2 | 1.67  (0.66 , 3.26) | 14.25  (7.77 , 23.84) | 752.1 | 11.21  (4.31 , 21.79) | 36.14  (0.89 , 58.98) | 222.5 | 0.45  (0.17 , 0.89) | 1.77  (0.95 , 2.84) | 291.2 |
|  | *Esophageal cancer* | 43.11  (7.44 , 97.19) | 258.93  (66.92 , 510.98) | 500.6 | 1.38  (0.23 , 3.1) | 8.67  (2.25 , 16.51) | 529.6 | 9.29  (1.56 , 20.95) | 21.39  (0.82 , 39.77) | 130.1 | 0.36  (0.06 , 0.82) | 0.98  (0.23 , 1.84) | 173.4 |
|  | *Gallbladder and biliary tract cancer* | 7.18  (1.3 , 16.51) | 36.42  (8.06 , 71.8) | 407.2 | 0.24  (0.04 , 0.55) | 1.28  (0.28 , 2.48) | 440.4 | 1.63  (0.28 , 3.77) | 3.33  (0.16 , 6.54) | 104.7 | 0.07  (0.01 , 0.16) | 0.17  (0.04 , 0.34) | 145 |
|  | *Kidney cancer* | 9.28  (3.42 , 18.61) | 130  (69 , 216.5) | 1301 | 0.28  (0.1 , 0.55) | 4.03  (2.15 , 6.62) | 1361 | 1.81  (0.64 , 3.68) | 9.77  (0.14 , 15.66) | 439.6 | 0.07  (0.02 , 0.14) | 0.44  (0.23 , 0.72) | 544 |
|  | *Leukemia* | 30.37  (11.18 , 60.79) | 195.01  (86.55 , 347.19) | 542.1 | 0.79  (0.29 , 1.56) | 4.89  (2.22 , 8.67) | 521.8 | 4.76  (1.76 , 9.6) | 10.01  (0.35 , 17.17) | 110.3 | 0.17  (0.06 , 0.35) | 0.42  (0.2 , 0.71) | 143.8 |
|  | *Liver cancer* | 79.66  (23.71 , 184.85) | 582.74  (209.11 , 1103.53) | 631.5 | 2.42  (0.7 , 5.49) | 17.55  (6.27 , 32.96) | 624.8 | 15.8  (4.35 , 36.03) | 38.82  (1.34 , 71.18) | 145.7 | 0.58  (0.16 , 1.34) | 1.58  (0.56 , 2.96) | 171.2 |
|  | *Multiple myeloma* | 6.25  (1.51 , 15.62) | 53.07  (14.8 , 117.17) | 748.7 | 0.2  (0.05 , 0.51) | 1.76  (0.48 , 3.71) | 761.4 | 1.41  (0.33 , 3.6) | 4.46  (0.15 , 9.42) | 215.2 | 0.06  (0.01 , 0.15) | 0.2  (0.06 , 0.41) | 255.3 |
|  | *Non-Hodgkin lymphoma* | 30.3  (9.59 , 66.52) | 204.95  (69.95 , 399.8) | 576.5 | 0.89  (0.28 , 1.97) | 5.88  (2.04 , 11.1) | 561.6 | 5.68  (1.77 , 12.61) | 13.44  (0.47 , 25.1) | 136.7 | 0.21  (0.06 , 0.47) | 0.56  (0.21 , 1.04) | 164.1 |
|  | *Pancreatic cancer* | 6.99  (-0.06 , 18.99) | 123.56  (-1.12 , 300.19) | 1668 | 0.23  (0 , 0.62) | 4.17  (-0.04 , 9.96) | 1745 | 1.53  (-0.01 , 4.28) | 10.27  (0.17 , 23.89) | 570.6 | 0.06  (0 , 0.17) | 0.49  (0 , 1.11) | 682.3 |
|  | *Thyroid cancer* | 2.94  (0.8 , 6.91) | 29.56  (9.18 , 59.24) | 903.9 | 0.08  (0.02 , 0.19) | 0.77  (0.24 , 1.49) | 854.5 | 0.54  (0.14 , 1.29) | 1.97  (0.05 , 3.75) | 264 | 0.02  (0.01 , 0.05) | 0.08  (0.03 , 0.16) | 321.6 |
| **Kuwait** | *Colon and rectum cancer* | 115.21  (65.83 , 173.22) | 883.51  (523.57 , 1336.85) | 666.9 | 3.6  (2.01 , 5.47) | 33.41  (19.33 , 50.9) | 827.2 | 23.3  (12.76 , 35.68) | 52.8  (1.52 , 80) | 126.6 | 0.98  (0.52 , 1.52) | 2.48  (1.4 , 3.83) | 152 |
|  | *Esophageal cancer* | 63.95  (14.54 , 115.01) | 254.22  (64.25 , 449.14) | 297.5 | 2.09  (0.48 , 3.8) | 10.08  (2.55 , 17.91) | 382.5 | 13.42  (3 , 24.58) | 14.83  (1.08 , 26.3) | 10.51 | 0.57  (0.13 , 1.08) | 0.74  (0.18 , 1.34) | 28.31 |
|  | *Gallbladder and biliary tract cancer* | 21.23  (4.26 , 41.7) | 63.84  (13.74 , 124.19) | 200.8 | 0.72  (0.14 , 1.42) | 2.48  (0.51 , 4.91) | 244.5 | 4.75  (0.96 , 9.43) | 3.84  (0.42 , 7.61) | -19.18 | 0.21  (0.04 , 0.42) | 0.18  (0.04 , 0.37) | -12.2 |
|  | *Kidney cancer* | 33.47  (17.8 , 51.65) | 227.26  (125.98 , 359.39) | 579 | 1.03  (0.53 , 1.6) | 7.83  (4.33 , 12.37) | 663.4 | 6.54  (3.31 , 10.25) | 12.5  (0.41 , 19.76) | 91.05 | 0.26  (0.13 , 0.41) | 0.53  (0.29 , 0.83) | 106.4 |
|  | *Leukemia* | 78.04  (38.45 , 127.39) | 225.95  (114.55 , 354.03) | 189.5 | 1.95  (0.95 , 3.21) | 6.9  (3.45 , 10.86) | 253.6 | 11.16  (5.35 , 18.54) | 10.97  (0.68 , 17.32) | -1.74 | 0.4  (0.19 , 0.68) | 0.44  (0.21 , 0.71) | 8.77 |
|  | *Liver cancer* | 96.3  (34.61 , 173.01) | 328.38  (127.07 , 608.71) | 241 | 2.99  (1.09 , 5.45) | 11.9  (4.58 , 22.06) | 298.1 | 18.74  (6.74 , 34.22) | 18.92  (1.34 , 34.91) | 0.94 | 0.73  (0.26 , 1.34) | 0.83  (0.32 , 1.54) | 13.41 |
|  | *Multiple myeloma* | 9.9  (2.95 , 19.54) | 49.82  (15.09 , 102.51) | 403.2 | 0.32  (0.09 , 0.64) | 1.76  (0.52 , 3.6) | 457.3 | 2.05  (0.58 , 4.21) | 2.77  (0.17 , 5.72) | 35.02 | 0.08  (0.02 , 0.17) | 0.12  (0.04 , 0.24) | 43.28 |
|  | *Non-Hodgkin lymphoma* | 57.22  (21.41 , 104.45) | 213.07  (79.27 , 391.07) | 272.4 | 1.56  (0.57 , 2.9) | 6.97  (2.52 , 12.61) | 345.7 | 9.34  (3.41 , 17.68) | 11.16  (0.68 , 20.56) | 19.49 | 0.35  (0.13 , 0.68) | 0.46  (0.16 , 0.85) | 30.79 |
|  | *Pancreatic cancer* | 28.08  (-0.21 , 66.23) | 206.95  (-1.8 , 472.86) | 637 | 0.94  (-0.01 , 2.23) | 8.06  (-0.07 , 18.73) | 757.5 | 6.12  (-0.05 , 14.69) | 12.42  (0.64 , 28.61) | 102.9 | 0.26  (0 , 0.64) | 0.59  (0 , 1.41) | 125.2 |
|  | *Thyroid cancer* | 9.53  (2.93 , 17.85) | 52.06  (17.62 , 93.61) | 446.1 | 0.27  (0.08 , 0.49) | 1.74  (0.59 , 3.06) | 551.2 | 1.79  (0.52 , 3.35) | 2.94  (0.13 , 5.24) | 64.29 | 0.07  (0.02 , 0.13) | 0.13  (0.04 , 0.23) | 83.34 |
| **Qatar** | *Colon and rectum cancer* | 33.1  (17.13 , 54.45) | 428.43  (244.83 , 665.56) | 1194 | 0.99  (0.51 , 1.64) | 13.13  (7.52 , 20.23) | 1226 | 35.06  (17.58 , 58.68) | 57.34  (2.88 , 86.69) | 63.54 | 1.67  (0.83 , 2.88) | 3.26  (1.88 , 4.94) | 94.89 |
|  | *Esophageal cancer* | 28.5  (6.15 , 55.02) | 237.35  (56.97 , 469.62) | 733 | 0.9  (0.2 , 1.76) | 7.69  (1.86 , 14.81) | 750.7 | 34.31  (7.57 , 65.58) | 35.02  (3.55 , 63.86) | 2.08 | 1.8  (0.39 , 3.55) | 2.2  (0.56 , 4.11) | 21.78 |
|  | *Gallbladder and biliary tract cancer* | 5.88  (1.27 , 11.83) | 41.75  (9.16 , 80.51) | 609.6 | 0.19  (0.04 , 0.38) | 1.35  (0.3 , 2.6) | 623.3 | 6.87  (1.42 , 13.96) | 5.56  (0.66 , 10.67) | -19.05 | 0.32  (0.07 , 0.66) | 0.31  (0.07 , 0.61) | -4.46 |
|  | *Kidney cancer* | 15.78  (7.59 , 27.59) | 201.78  (109.1 , 330.15) | 1179 | 0.5  (0.24 , 0.87) | 6.45  (3.35 , 10.55) | 1190 | 18.56  (8.5 , 32.64) | 26.88  (1.53 , 43.32) | 44.82 | 0.88  (0.4 , 1.53) | 1.48  (0.75 , 2.4) | 68.57 |
|  | *Leukemia* | 24.41  (10.5 , 44.98) | 222.31  (102.03 , 419.63) | 810.7 | 0.6  (0.26 , 1.05) | 5.43  (2.55 , 9.82) | 807.5 | 16.7  (7.29 , 28.66) | 18.84  (1.41 , 32.8) | 12.79 | 0.78  (0.32 , 1.41) | 1.06  (0.48 , 1.92) | 35.46 |
|  | *Liver cancer* | 80.61  (28.17 , 155.98) | 731.91  (284.14 , 1373.34) | 807.9 | 2.6  (0.91 , 5.05) | 24.36  (9.42 , 44.75) | 836.3 | 94.63  (32.54 , 185.21) | 94.04  (8.56 , 169.89) | -0.62 | 4.38  (1.51 , 8.56) | 4.97  (1.99 , 8.97) | 13.65 |
|  | *Multiple myeloma* | 3.13  (0.86 , 6.66) | 37.63  (10.59 , 76.97) | 1102 | 0.1  (0.03 , 0.21) | 1.18  (0.32 , 2.49) | 1134 | 3.32  (0.9 , 7.33) | 4.47  (0.32 , 9.76) | 34.47 | 0.15  (0.04 , 0.32) | 0.22  (0.06 , 0.49) | 50.01 |
|  | *Non-Hodgkin lymphoma* | 14.77  (5.46 , 28.31) | 149.79  (56.01 , 288.73) | 914.1 | 0.41  (0.15 , 0.77) | 3.96  (1.48 , 7.6) | 873.4 | 12.79  (4.74 , 24.33) | 14.33  (1.13 , 27.24) | 12.07 | 0.59  (0.22 , 1.13) | 0.73  (0.28 , 1.39) | 24.41 |
|  | *Pancreatic cancer* | 7.11  (-0.06 , 16.89) | 118.23  (-1.04 , 276.5) | 1563 | 0.23  (0 , 0.54) | 3.84  (-0.03 , 8.89) | 1587 | 8.14  (-0.07 , 19.78) | 14.52  (0.93 , 33.53) | 78.28 | 0.38  (0 , 0.93) | 0.79  (-0.01 , 1.82) | 105.4 |
|  | *Thyroid cancer* | 2.3  (0.69 , 4.63) | 25.38  (8.34 , 47.68) | 1002 | 0.06  (0.02 , 0.13) | 0.63  (0.19 , 1.15) | 889.8 | 2.08  (0.62 , 4.26) | 2.46  (0.19 , 4.53) | 18.12 | 0.09  (0.03 , 0.19) | 0.12  (0.04 , 0.22) | 30.71 |
| **United Arab Emirates** | *Colon and rectum cancer* | 191.42  (86 , 360.06) | 2916.11  (1668.59 , 4453.84) | 1423 | 5.64  (2.58 , 10.5) | 82.37  (46.98 , 124.16) | 1361 | 55.13  (25 , 99.19) | 79.56  (4.96 , 122.47) | 44.31 | 2.75  (1.22 , 4.96) | 4.14  (2.28 , 6.46) | 50.72 |
|  | *Esophageal cancer* | 280.53  (38.21 , 600.15) | 5360.69  (869.24 , 11037.27) | 1811 | 7.8  (1.02 , 16.97) | 144.89  (24.08 , 302.51) | 1758 | 61.69  (7.34 , 137.8) | 102.3  (5.87 , 216.19) | 65.83 | 2.53  (0.29 , 5.87) | 4.29  (0.65 , 9.19) | 69.19 |
|  | *Gallbladder and biliary tract cancer* | 29.7  (5.63 , 63.8) | 420.71  (78.12 , 835.05) | 1317 | 0.82  (0.16 , 1.78) | 11.21  (2.09 , 22.27) | 1262 | 6.56  (1.21 , 14.41) | 7.93  (0.62 , 15.88) | 20.9 | 0.28  (0.05 , 0.62) | 0.34  (0.06 , 0.67) | 21.13 |
|  | *Kidney cancer* | 104.6  (32.55 , 291.49) | 2904.99  (960 , 5411.02) | 2677 | 2.72  (0.81 , 7.67) | 74.62  (23.55 , 141.81) | 2648 | 19.43  (5.03 , 56.15) | 48.57  (2.2 , 90.48) | 149.9 | 0.75  (0.18 , 2.2) | 1.88  (0.5 , 3.53) | 149.6 |
|  | *Leukemia* | 95.56  (42.99 , 176.19) | 1245.04  (604.96 , 2193.76) | 1203 | 2.27  (1.04 , 4.17) | 30.3  (14.9 , 51.85) | 1237 | 15.06  (6.44 , 28.03) | 20.36  (1.14 , 34.16) | 35.2 | 0.6  (0.24 , 1.14) | 0.8  (0.36 , 1.37) | 33.8 |
|  | *Liver cancer* | 135.34  (33.82 , 311.24) | 2335.46  (580.57 , 6315.62) | 1626 | 3.67  (0.87 , 8.83) | 62.72  (15.17 , 171.17) | 1608 | 28.28  (6.72 , 74.4) | 44.66  (3.1 , 130.37) | 57.93 | 1.15  (0.28 , 3.1) | 1.86  (0.42 , 5.49) | 60.69 |
|  | *Multiple myeloma* | 23.8  (5.78 , 55.62) | 412.56  (96.3 , 1076.35) | 1634 | 0.63  (0.15 , 1.51) | 10.71  (2.58 , 28.3) | 1596 | 4.69  (1.09 , 12.09) | 7.15  (0.48 , 18.86) | 52.42 | 0.18  (0.04 , 0.48) | 0.28  (0.06 , 0.75) | 52.07 |
|  | *Non-Hodgkin lymphoma* | 117.31  (35.07 , 280.96) | 1576.06  (508.94 , 3189.58) | 1244 | 2.69  (0.8 , 6.63) | 36.82  (11.49 , 74.39) | 1267 | 15.06  (4.17 , 38.75) | 20.41  (1.37 , 42.06) | 35.55 | 0.53  (0.14 , 1.37) | 0.69  (0.2 , 1.45) | 30.05 |
|  | *Pancreatic cancer* | 67.05  (-0.58 , 184.02) | 2504.21  (-22.73 , 6537.22) | 3635 | 1.87  (-0.02 , 5.12) | 68.33  (-0.62 , 176.66) | 3545 | 14.99  (-0.12 , 41.88) | 48.51  (1.73 , 122.46) | 223.7 | 0.61  (-0.01 , 1.73) | 2.01  (-0.02 , 5.04) | 227.4 |
|  | *Thyroid cancer* | 20.63  (5.59 , 43.43) | 468.13  (131.04 , 948.18) | 2169 | 0.49  (0.13 , 1.05) | 10.64  (2.96 , 21.49) | 2073 | 3.37  (0.82 , 7.67) | 7  (0.29 , 14.59) | 107.5 | 0.13  (0.03 , 0.29) | 0.25  (0.07 , 0.51) | 96.73 |

*BMI, body mass index; GCC, gulf cooperation council; ASDR, age-standardized DALYs rate; ASMR, age-standardized mortality rate; DALYs, disability-adjusted life-years; UI, uncertainty interval*

**Supplementary Table 3A: Burden of DALYs and Deaths of cancer attributable to high BMI from 1990 to 2019 in GCC countries by years.**

| **Year** | **Number of DALYs** | | | | | | **Number of Deaths** | | | | | |
| --- | --- | --- | --- | --- | --- | --- | --- | --- | --- | --- | --- | --- |
|  | **Saudi Arabia** | **Bahrain** | **Oman** | **Kuwait** | **Qatar** | **United Arab Emirates** | **Saudi Arabia** | **Bahrain** | **Oman** | **Kuwait** | **Qatar** | **United Arab Emirates** |
| **1990** | 5632.89 | 354.31 | 482.90 | 865.11 | 292.69 | 1452.15 | 199.63 | 12.63 | 15.99 | 29.36 | 9.84 | 41.65 |
| **1991** | 6269.40 | 375.61 | 530.64 | 793.80 | 309.55 | 1597.65 | 222.37 | 13.46 | 17.59 | 26.93 | 10.47 | 45.73 |
| **1992** | 6909.14 | 394.67 | 580.81 | 702.67 | 331.60 | 1744.78 | 245.52 | 14.18 | 19.25 | 24.32 | 11.22 | 49.87 |
| **1993** | 7602.61 | 408.97 | 626.90 | 725.60 | 356.34 | 1906.31 | 270.69 | 14.68 | 20.85 | 25.27 | 12.05 | 54.45 |
| **1994** | 8365.96 | 423.23 | 675.11 | 758.51 | 367.69 | 2084.59 | 298.49 | 15.22 | 22.57 | 26.87 | 12.09 | 59.94 |
| **1995** | 9154.96 | 437.76 | 725.90 | 784.61 | 382.81 | 2265.39 | 327.47 | 15.80 | 24.37 | 28.13 | 12.39 | 65.01 |
| **1996** | 9953.36 | 447.42 | 798.45 | 823.85 | 423.94 | 2441.02 | 357.38 | 16.30 | 26.73 | 30.00 | 14.17 | 69.96 |
| **1997** | 10750.17 | 447.96 | 867.43 | 958.42 | 463.61 | 2596.65 | 387.82 | 16.43 | 28.97 | 34.94 | 15.70 | 74.33 |
| **1998** | 11547.37 | 457.83 | 918.99 | 1018.32 | 498.39 | 2758.44 | 418.29 | 16.98 | 30.87 | 37.38 | 16.89 | 78.82 |
| **1999** | 12390.23 | 473.89 | 958.58 | 1163.30 | 531.59 | 2955.27 | 449.79 | 17.63 | 32.56 | 42.68 | 17.93 | 84.31 |
| **2000** | 13163.66 | 470.58 | 1025.45 | 1234.20 | 561.57 | 3183.56 | 478.41 | 17.23 | 35.03 | 45.57 | 18.80 | 91.21 |
| **2001** | 13803.49 | 464.73 | 1125.76 | 1200.02 | 593.55 | 3428.85 | 502.15 | 16.77 | 38.14 | 45.54 | 19.89 | 97.94 |
| **2002** | 14347.78 | 514.78 | 1226.11 | 1316.41 | 652.38 | 3738.45 | 521.19 | 19.18 | 41.35 | 49.90 | 21.87 | 106.38 |
| **2003** | 14913.78 | 584.35 | 1290.41 | 1368.32 | 721.19 | 4081.68 | 539.58 | 21.96 | 43.77 | 51.87 | 24.02 | 116.93 |
| **2004** | 15373.82 | 637.94 | 1324.67 | 1433.49 | 782.28 | 4519.65 | 553.35 | 23.75 | 45.37 | 54.58 | 25.83 | 130.39 |
| **2005** | 16073.83 | 680.66 | 1415.72 | 1557.73 | 863.08 | 4979.37 | 571.01 | 25.13 | 48.91 | 59.03 | 28.31 | 143.40 |
| **2006** | 16796.74 | 705.23 | 1590.68 | 1711.82 | 943.96 | 5597.38 | 591.13 | 25.87 | 54.73 | 64.96 | 31.11 | 158.52 |
| **2007** | 17723.61 | 740.60 | 1784.23 | 1841.39 | 1054.53 | 6386.30 | 618.22 | 26.95 | 60.74 | 70.21 | 34.68 | 174.29 |
| **2008** | 18723.27 | 795.89 | 1938.38 | 2150.50 | 1168.59 | 7372.91 | 647.35 | 28.63 | 65.98 | 82.70 | 37.96 | 195.25 |
| **2009** | 19666.58 | 862.31 | 2055.30 | 2196.16 | 1306.32 | 8504.50 | 675.27 | 30.58 | 70.59 | 84.93 | 41.73 | 221.38 |
| **2010** | 20903.33 | 940.56 | 2168.13 | 2136.43 | 1460.98 | 9483.15 | 711.52 | 32.89 | 74.57 | 82.52 | 45.87 | 247.00 |
| **2011** | 21745.82 | 999.78 | 2444.68 | 2175.17 | 1561.99 | 10532.25 | 734.23 | 34.54 | 82.70 | 84.81 | 48.98 | 275.18 |
| **2012** | 22848.64 | 1050.47 | 2722.37 | 2343.66 | 1664.82 | 11652.09 | 765.83 | 36.10 | 90.65 | 91.18 | 52.32 | 305.85 |
| **2013** | 24419.84 | 1092.80 | 2767.67 | 2474.54 | 1802.15 | 12961.76 | 811.61 | 37.53 | 92.65 | 96.27 | 56.77 | 340.12 |
| **2014** | 26179.86 | 1154.63 | 2781.43 | 2622.22 | 1943.42 | 13979.33 | 864.28 | 39.77 | 93.82 | 101.61 | 61.72 | 363.44 |
| **2015** | 27837.37 | 1235.65 | 2890.07 | 2804.98 | 2014.33 | 15468.79 | 912.76 | 42.95 | 98.04 | 107.41 | 64.49 | 405.79 |
| **2016** | 29875.44 | 1328.05 | 3025.68 | 3031.92 | 2140.65 | 17028.34 | 973.56 | 46.62 | 102.92 | 116.23 | 69.30 | 451.10 |
| **2017** | 31917.72 | 1446.90 | 3139.66 | 3285.63 | 2279.30 | 18732.92 | 1033.98 | 51.18 | 106.46 | 125.47 | 74.56 | 501.26 |
| **2018** | 34157.91 | 1601.96 | 3130.23 | 3570.28 | 2489.10 | 20733.04 | 1102.14 | 56.67 | 105.51 | 136.20 | 81.96 | 560.47 |
| **2019** | 36554.20 | 1769.26 | 3157.86 | 3870.31 | 2699.02 | 22934.50 | 1176.87 | 62.99 | 106.28 | 147.58 | 89.92 | 626.52 |

*BMI, body mass index; GCC, gulf cooperation council; ASDR, age-standardized DALYs rate; ASMR, age-standardized mortality rate; DALYs, disability-adjusted life-years.*

**Cont. Supplementary Table 3B: Burden of DALYs and Deaths of cancer attributable to high BMI from 1990 to 2019 in GCC countries by years.**

| **Year** | **ASMR per 100,000** | | | | | | **ASDR per 100,000** | | | | | |
| --- | --- | --- | --- | --- | --- | --- | --- | --- | --- | --- | --- | --- |
|  | **Saudi Arabia** | **Bahrain** | **Oman** | **Kuwait** | **Qatar** | **United Arab Emirates** | **Saudi Arabia** | **Bahrain** | **Oman** | **Kuwait** | **Qatar** | **United Arab Emirates** |
| **1990** | 3.57 | 8.09 | 2.40 | 5.20 | 11.40 | 9.89 | 85.77 | 181.29 | 61.06 | 122.11 | 242.93 | 235.58 |
| **1991** | 3.85 | 8.39 | 2.56 | 4.51 | 11.73 | 10.21 | 91.90 | 187.63 | 65.05 | 105.96 | 248.58 | 242.66 |
| **1992** | 4.10 | 8.56 | 2.73 | 4.00 | 12.07 | 10.49 | 97.48 | 191.78 | 69.20 | 93.25 | 254.95 | 248.59 |
| **1993** | 4.36 | 8.53 | 2.90 | 4.01 | 12.40 | 10.79 | 103.13 | 192.16 | 73.11 | 93.65 | 260.98 | 254.76 |
| **1994** | 4.64 | 8.54 | 3.07 | 4.18 | 11.26 | 11.43 | 109.18 | 192.64 | 77.47 | 97.51 | 240.82 | 266.73 |
| **1995** | 4.92 | 8.64 | 3.25 | 4.25 | 10.56 | 11.71 | 115.18 | 194.10 | 81.66 | 99.57 | 229.08 | 272.41 |
| **1996** | 5.20 | 8.77 | 3.45 | 4.32 | 12.09 | 12.01 | 121.14 | 195.32 | 86.62 | 100.51 | 257.29 | 278.75 |
| **1997** | 5.46 | 8.68 | 3.66 | 4.80 | 13.10 | 12.18 | 126.66 | 191.67 | 91.53 | 111.90 | 275.65 | 282.07 |
| **1998** | 5.70 | 8.81 | 3.85 | 4.90 | 13.45 | 12.31 | 131.55 | 192.73 | 96.03 | 113.43 | 281.69 | 284.55 |
| **1999** | 5.92 | 8.88 | 4.05 | 5.40 | 13.44 | 12.56 | 136.30 | 193.45 | 100.51 | 127.19 | 281.24 | 289.43 |
| **2000** | 6.10 | 8.23 | 4.28 | 5.61 | 13.06 | 13.19 | 139.90 | 180.85 | 106.07 | 132.72 | 275.41 | 301.91 |
| **2001** | 6.24 | 7.56 | 4.48 | 5.41 | 13.08 | 13.55 | 142.95 | 167.73 | 111.21 | 125.59 | 274.47 | 309.47 |
| **2002** | 6.30 | 8.47 | 4.73 | 5.69 | 13.62 | 14.00 | 144.35 | 182.95 | 117.38 | 132.47 | 283.93 | 319.09 |
| **2003** | 6.33 | 9.31 | 4.98 | 5.60 | 13.90 | 14.99 | 145.19 | 197.87 | 123.69 | 130.59 | 289.28 | 337.92 |
| **2004** | 6.31 | 9.50 | 5.19 | 5.67 | 13.72 | 16.30 | 144.68 | 200.49 | 129.13 | 131.46 | 286.50 | 362.87 |
| **2005** | 6.29 | 9.47 | 5.54 | 5.88 | 14.15 | 16.94 | 145.14 | 197.66 | 137.30 | 136.29 | 293.75 | 375.56 |
| **2006** | 6.34 | 9.19 | 5.95 | 6.14 | 15.12 | 17.29 | 146.70 | 190.51 | 146.82 | 142.21 | 307.68 | 383.55 |
| **2007** | 6.45 | 8.95 | 6.35 | 6.33 | 16.17 | 16.95 | 149.43 | 184.47 | 156.69 | 145.66 | 323.01 | 377.78 |
| **2008** | 6.55 | 8.79 | 6.77 | 7.05 | 16.36 | 17.06 | 151.91 | 180.72 | 166.91 | 159.84 | 324.98 | 380.34 |
| **2009** | 6.63 | 8.62 | 7.15 | 6.97 | 16.43 | 17.30 | 153.53 | 177.10 | 174.87 | 156.45 | 325.26 | 386.41 |
| **2010** | 6.75 | 8.51 | 7.23 | 6.50 | 16.45 | 17.36 | 156.30 | 174.50 | 174.37 | 145.11 | 324.40 | 388.94 |
| **2011** | 6.73 | 8.31 | 7.20 | 6.30 | 16.34 | 17.38 | 155.89 | 169.45 | 172.05 | 139.41 | 318.67 | 390.32 |
| **2012** | 6.75 | 8.13 | 7.12 | 6.37 | 16.20 | 17.34 | 156.38 | 164.19 | 169.61 | 140.34 | 313.03 | 390.26 |
| **2013** | 6.82 | 7.78 | 7.12 | 6.30 | 16.21 | 16.88 | 158.55 | 157.36 | 164.76 | 138.24 | 310.24 | 383.16 |
| **2014** | 6.91 | 7.52 | 7.15 | 6.28 | 16.57 | 14.95 | 161.09 | 153.26 | 161.81 | 137.40 | 310.62 | 347.33 |
| **2015** | 6.92 | 7.48 | 7.26 | 6.22 | 17.18 | 15.00 | 161.94 | 152.71 | 162.05 | 136.38 | 306.32 | 349.63 |
| **2016** | 7.01 | 7.60 | 7.30 | 6.28 | 17.22 | 15.00 | 164.56 | 154.68 | 160.55 | 137.44 | 304.59 | 350.59 |
| **2017** | 7.05 | 7.77 | 7.26 | 6.34 | 16.95 | 14.98 | 165.93 | 157.89 | 159.67 | 138.58 | 299.98 | 351.04 |
| **2018** | 7.09 | 7.86 | 6.97 | 6.41 | 16.40 | 15.08 | 167.53 | 160.77 | 155.43 | 140.01 | 294.76 | 354.24 |
| **2019** | 7.14 | 8.04 | 6.87 | 6.54 | 16.13 | 15.24 | 169.13 | 164.69 | 153.97 | 141.68 | 291.55 | 358.50 |

*BMI, body mass index; GCC, gulf cooperation council; ASDR, age-standardized DALYs rate; ASMR, age-standardized mortality rate; DALYs, disability-adjusted life-years.*
